# Selective sodium-glucose cotransporter-2 inhibitors in the improvement of hemoglobin and hematocrit in patients with type 2 diabetes mellitus: a network meta-analysis

**DOI:** 10.3389/fendo.2024.1333624

**Published:** 2024-02-01

**Authors:** Yuanyuan Luo, Ruojing Bai, Wei Zhang, Guijun Qin

**Affiliations:** ^1^ Department of Endocrinology and Metabolism, The First Affiliated Hospital of Zhengzhou University, Zhengzhou University, Zhengzhou, Henan, China; ^2^ Department of Geriatric Medicine, Beijing Tsinghua Changgung Hospital, School of Clinical Medicine, Tsinghua University, Beijing, China

**Keywords:** selective sodium-glucose cotransporter-2 inhibitor, type 2 diabetes mellitus, network meta, hemoglobin, hematocrit

## Abstract

**Objective:**

To compare the effects of different selective sodium-glucose cotransporter-2 inhibitors (SGLT2i) on hemoglobin and hematocrit in patients with type 2 diabetes mellitus (T2DM) with a network meta-analysis (NMA).

**Methods:**

Randomized controlled trials (RCTs) on SGLT2i for patients with T2DM were searched in PubMed, Embase, Cochrane Library, and Web of Science from inception of these databases to July 1, 2023. The risk of bias (RoB) tool was used to evaluate the quality of the included studies, and R software was adopted for data analysis.

**Results:**

Twenty-two articles were included, involving a total of 14,001 T2DM patients. SGLT2i included empagliflozin, dapagliflozin, and canagliflozin. The NMA results showed that compared with placebo, canagliflozin 100mg, canagliflozin 300mg, dapagliflozin 10mg, dapagliflozin 2mg, dapagliflozin 50mg, dapagliflozin 5mg, empagliflozin 25mg, and dapagliflozin 20mg increased hematocrit in patients with T2DM, while canagliflozin 100mg, canagliflozin 200mg, canagliflozin 300mg increased hemoglobin in patients with T2DM. In addition, the NMA results indicated that canagliflozin 100mg had the best effect on the improvement of hematocrit, and canagliflozin 200mg had the best effect on the improvement of hemoglobin.

**Conclusion:**

Based on the existing studies, we concluded that SGLT2i could increase hematocrit and hemoglobin levels in patients with T2DM, and canagliflozin 100mg had the best effect on the improvement of hematocrit, while canagliflozin 200mg had the best effect on the improvement of hemoglobin.

**Systematic review registration:**

https://www.crd.york.ac.uk/PROSPERO/#loginpage, identifier PROSPERO (CRD42023477103).

## 1 Introduction

In recent decades, the prevalence of type 2 diabetes mellitus (T2DM) has increased rapidly. According to the prediction of the International Diabetes Federation (IDF), there will be over 700 million T2DM cases by 2045, making the disease a significant “killer” that threatens human health in the 21st century (1, 2). The global prevalence of diabetes was estimated to be 10.5% in adults aged 20-79 in 2021 (536.6 million cases). This number is predicted to rise according to a diabetes mellitus (DM) prediction model of IDF (3). In addition, anemia is prevalent in patients with T2DM, with approximately 1/4 of T2DM patients being anemic (4). Early evidence suggests that the development of anemia in patients with DM is usually related to nephropathy, with erythropoietin (EPO) deficiency or iron deficiency being the main causes (5, 6). Anemia is an important cause of irreversible damage to heart, brain, kidney, and other important organs in patients with T2DM, as well as an important cause of an increased mortality rate. It is an independent risk factor affecting the prognosis in patients with T2DM (7). Therefore, how the anemia status of T2DM patients can be improved has become a hot topic (8).

At present, a comprehensive management model is adopted for the treatment of T2DM, involving lowering blood glucose, lowering blood pressure, regulating blood lipids, anti-platelet, weight control and improving lifestyle. Despite a comprehensive management, the blood glucose in some patients is not well controlled (9). Sodium-glucose cotransporter-2 inhibitor (SGLT2i) is a hot topic in recent research, mainly focusing on the selective SGLT2i. SGLT2 is a glucose transporter in the renal proximal tubule (10). In recent years, it has been found that SGLT2 can ensure the balance between filtration and renal reabsorption of glucose, without urinary glucose excretion. A large number of studies have pointed out that by inhibiting SGLT2, the reabsorption of glucose in the kidney can be reduced, increasing the urinary glucose excretion. In this way, the blood glucose level can be reduced (11, 12). At present, SGLT2i mainly includes empagliflozin, dapagliflozin, and canagliflozin. When SGLT2i is used to treat diabetes, it can reduce blood glucose separately, without relying on insulin. At the same time, it can effectively reduce the weight of patients, reduce the blood pressure level, and reduce urinary protein, helping patients with DM reach the blood control goal and protect kidney functions effectively (13, 14). A previous meta-analysis (15) indicated that SGLT2i improved hemoglobin and hematocrit in patients with T2DM. However, the effect of SGLT2i on the improvement of hemoglobin and hematocrit in patients with T2DM remains controversial. Some studies indicate that it can increase hemoglobin and hematocrit in patients with T2DM, while other studies show the opposite results (16, 17). In addition, there is a lack of head-to-head comparison of several types of SGLT2i, and the recommended dosage also remains controversial (18). Therefore, a network meta-analysis (NMA) was performed in this study to address the above controversies and provide new options for the treatment of patients with T2DM.

## 2 Methods

### 2.1 Meta registration

This meta-analysis was conducted in accordance with the PRISMA (Preferred Reporting Items for Systematic Reviews and Meta-Analyses) statement. The protocol for the study has been registered with PROSPERO (registration number: CRD42023477103).

### 2.2 Literature search

Cochrane, PubMed, Embase, and Web of Science were searched for randomized controlled trials (RCTs) on the effects of SGLT2i on the improvement of hemoglobin and hematocrit in patients with T2DM from the inception of these databases up to July 1, 2023. A combination of subject terms and free words were used for literature search, including type 2 diabetes mellitus, sodium-glucose cotransporter-2, empagliflozin, dapagliflozin, canagliflozin, hemoglobin, and hematocrit. The detailed search strategy is shown in **Supplementary Material 1**.

### 2.3 Eligibility criteria

Inclusion criteria: The participants were adults who met the diagnostic criteria (19) of T2DM (HbA1c levels ≥ 7.0% to ≤10.0%) in the included studies. The intervention was SGLT2i (empagliflozin, dapagliflozin, canagliflozin), and the control intervention was placebo. The primary outcome was hematocrit, and the secondary outcome was hemoglobin. The study type was an RCT.

Exclusion criteria: duplicate publications, animal studies, case reports, conference abstracts, reviews, studies with no available full texts.

### 2.4 Data extraction

Two independent authors performed a rigorous screening of literature based on the pre-defined inclusion and exclusion criteria. In case of any disagreement, a consensus was reached through discussion between the two authors or consultation with a third researcher. The following key data were extracted from the included studies: first author, year of publication, country, sample size, gender, age, interventions, and outcome measures.

### 2.5 Quality assessment

The risk of bias was assessed using version 2 of the Cochrane risk-of-bias tool for randomized trials (RoB 2) (20) from five major domains: bias arising from the randomization process, bias due to deviations from intended interventions, bias due to missing outcome data, bias in the measurement of the outcome, and bias in the selection of the reported results. The judgements can be “low risk of bias”, “some concerns” and “high risk of bias”. The results of the study were checked by the two researchers and disagreements, if any, were discussed between the two researchers, or addressed through consultation with a third researcher.

### 2.6 Quality rating by GRADE

The Grading of Recommendations Assessment, Development and Evaluation (GRADE) system was adopted to assess the methodological quality of the evidence to determine the quality of the results (21). We considered five domains that may reduce the quality of evidence, including study limitations, inconsistency, indirectness, imprecision or wide confidence intervals, and publication bias. In addition, we reviewed factors that could reduce the quality of evidence, namely effect size, possible confounders, and dose-effect relationship. A comprehensive description of the quality of the evidence for each outcome is provided in **Table 1**.

**Table 1:** Quality rating by GRADE.

Outcome	Grade
Hemoglobin	Low
Hematocrit	Moderate

### 2.7 Data analysis

R4.3.2 software (R Foundation for Statistical Computing) was used to conduct Bayesian NMA of multi-group trials using a Markov chain Monte Carlo method with vague prior distribution and a random effects model (22)to obtain the best pooled estimates and probabilities for each treatment regimen. The trajectory plot and Brooks-Gelman-Rubin plot were plotted to evaluate the convergence of the model, and the continuous outcomes were expressed as posterior mean difference (MD) and its 95% confidence interval (CI). The surface under the cumulative ranking curve (SUCRA) was calculated to estimate the probability of each treatment as the optimal intervention. The network, “networkplot” and “sencode” packages in STATA 15.0 were used to draw network diagram and funnel plots. In the network diagram, each circle represents a drug, and the lines represent existing comparisons. The size of the circle is positively related to the number of included patients. The “ggplot2” package was used to create the cumulative probability plot, and a *P-value* < 0.05 was statistically significant.

## 3 Results

### 3.1 Literature screening process and results

**Figure 1:**
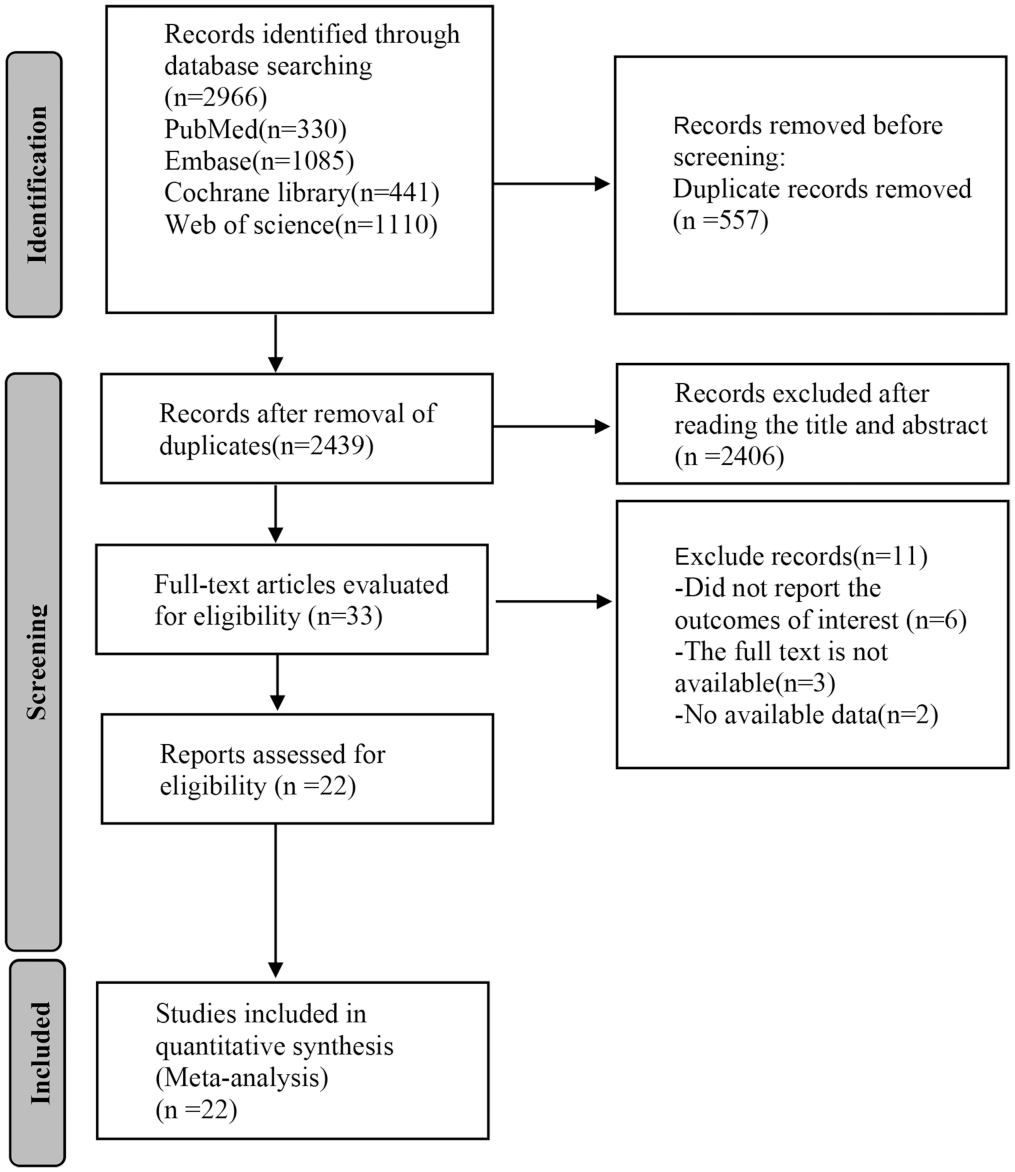
Flow chart of literature search.

A total of 2,966 articles were obtained through the preliminary search of databases, and 577 duplicates were removed. After reading the titles and abstracts, we excluded 2,406 articles, and 11 articles were eliminated by reading the full texts. A total of 22 (23–44) articles were eventually included for analysis (**Figure 1**).

### 3.2 Characteristics of literature and risk of bias assessment

**Figure 2:**
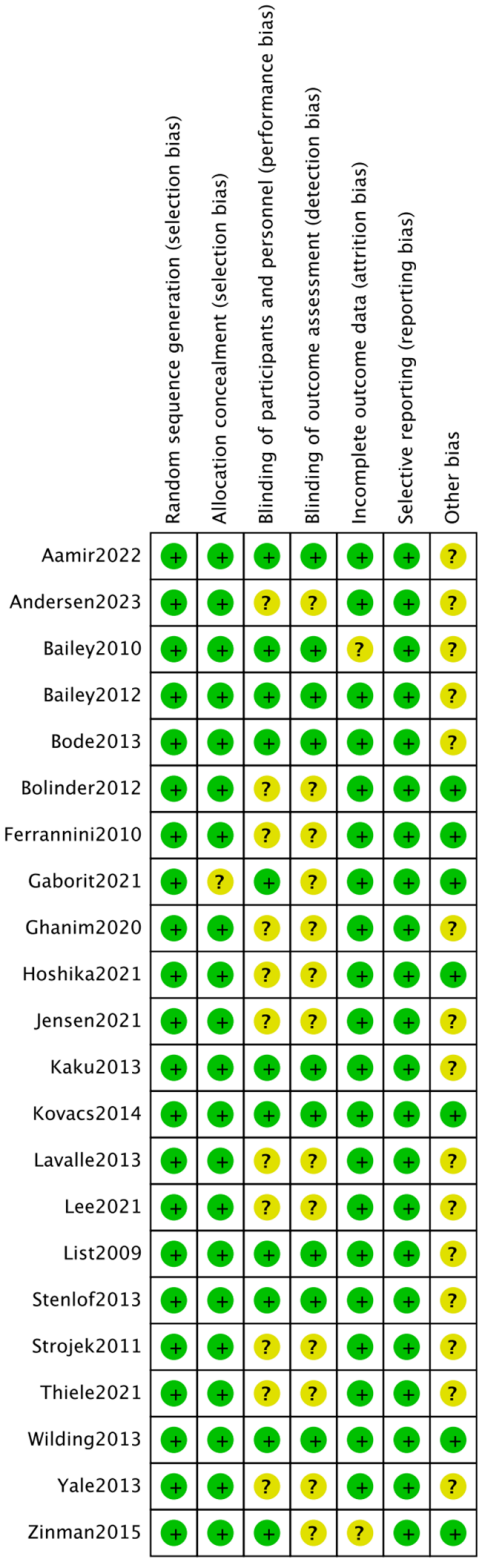
Risk assessment of literature bias. The quality assessment for each bias risk item is presented as a percentage. “+” means low risk and “?” means unclear.

**Figure 3:**
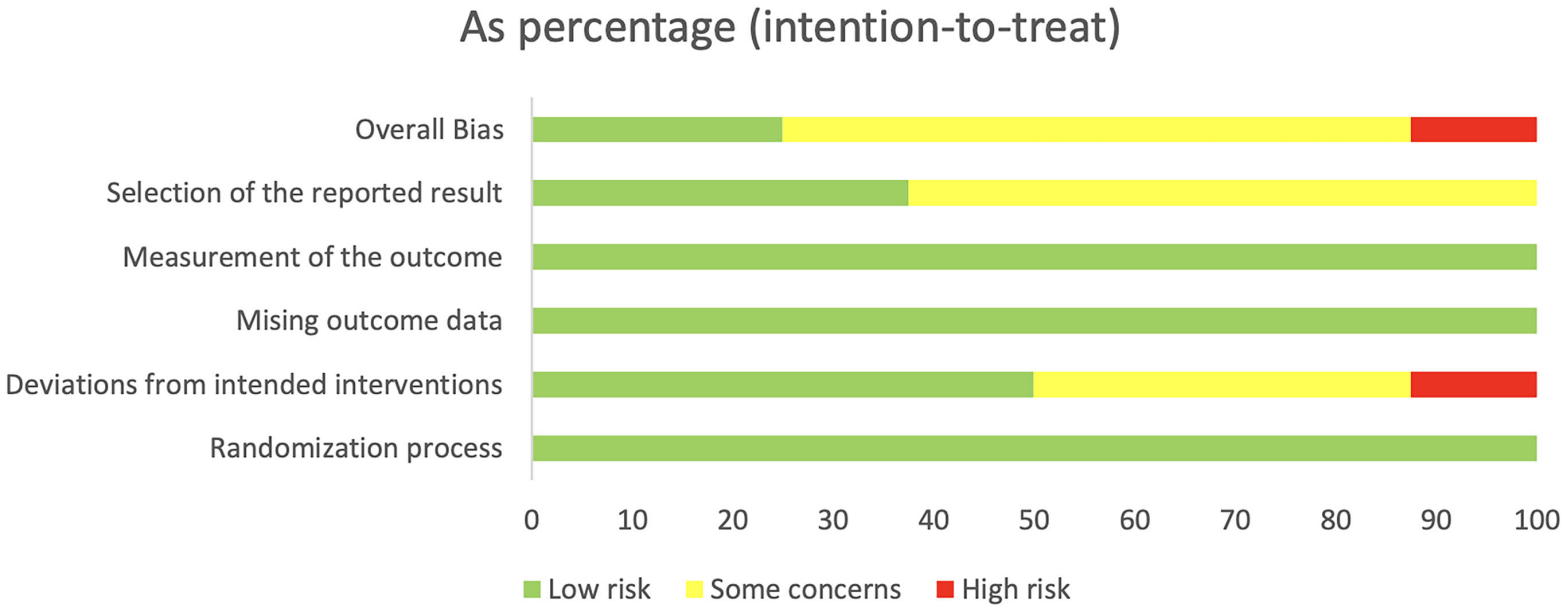
Risk assessment of literature bias. Quality assessment of each bias risk item for each study. Each color represents a difference in the risk of bias: green represents low risk of bias, yellow represents unclear risk, and red represents high risk of bias.

The 22 articles involved 14,001 T2DM patients, and the mean age was 50-64 years. The SGLT2i covered empagliflozin, dapagliflozin, and canagliflozin, with a dose range of 1 to 300 mg. The specific characteristics of literature are shown in **Table 2**. All the included studies clearly described the blinding method, and the high risk mainly arose from deviations from intended interventions. **Figures 2**, **3** show the risk of bias assessment of the included studies.

**Table 2:** Characteristics of the included studies.

Study	Year	Country	Sample size	Gender (M/F)	Mean age (years)	Intervention	Outcome
Aamir	2022	Pakistan	Empagliflozin:129 Placebo:115	125/119	Empagliflozin:50.1 Placebo:50	Empagliflozin10mg once/daily	F1; F2;
Andersen	2023	Denmark	Empagliflozin:95 Placebo:95	162/28	Empagliflozin:65 Placebo:63	Empagliflozin 10mg once/daily	F1
Bailey	2010	England	Dapagliflozin2.5mg:137 Dapagliflozin5mg:137 Dapagliflozin10mg:135 Placebo:137	292/254	Dapagliflozin2.5mg:55 Dapagliflozin5mg:54.3 Dapagliflozin10mg:52.7 Placebo:53.7	Dapagliflozin (2.5mg, 5mg, 10mg once/daily)	F2
Bailey	2012	England	Dapagliflozin1mg:68 Dapagliflozin2.5mg:72 Dapagliflozin5mg:74 Placebo:68	142/140	Dapagliflozin1mg:53.7 Dapagliflozin2.5mg:53.5 Dapagliflozin5mg:51.3 Placebo:53.5	Dapagliflozin (2.5mg, 1mg, 5mg once/daily)	F2
Bode	2013	USA	Canagliflozin100mg:241 Canagliflozin200mg:236 Placebo:237	396/318	Canagliflozin100mg:64.3 Canagliflozin200mg:63.4 Placebo:63.2	Canagliflozin(100mg,200mg once/daily)	F1
Bolinder	2012	Sweden	Dapagliflozin:89 Placebo:91	100/80	Dapagliflozin:60.6 Placebo:60.8	Dapagliflozin10mg once/daily	F2
Ferrannini	2010	Mexico	Dapagliflozin2.5mg:65 Dapagliflozin5mg:64 Dapagliflozin10mg:70 Placebo:75	132/172	Dapagliflozin2.5mg:53 Dapagliflozin5mg:52.6 Dapagliflozin10mg:50.6 Placebo:52.7	Dapagliflozin (2.5mg, 5mg, 10mg once/daily)	F2
Gaborit	2021	France	Empagliflozin:26 Placebo:25	20/31	Empagliflozin:57 Placebo:58.6	Empagliflozin 10mg once/daily	F2
Ghanim	2020	USA	Dapagliflozin:24 Placebo:23	NA	Dapagliflozin:61 Placebo:62	Dapagliflozin10mg once/daily	F1, F2
Hoshika	2021	Japan	Empagliflozin:46 Placebo:50	77/19	Empagliflozin:63.9 Placebo:64.6	Empagliflozin 10mg once/daily	F2
Jensen	2021	Denmark	Empagliflozin:60 Placebo:60	99/21	Empagliflozin:68 Placebo:67	Empagliflozin 10mg once/daily	F1, F2
Kaku	2013	Japan	Dapagliflozin1mg:59 Dapagliflozin2.5mg:56 Dapagliflozin5mg:58 Dapagliflozin10mg:52 Placebo:54	215/64	Dapagliflozin1mg:55.9 Dapagliflozin2.5mg:57.7 Dapagliflozin5mg:58 Dapagliflozin10mg:56.5 Placebo:58.4	Dapagliflozin (1mg, 2.5mg, 1mg, 5mg once/daily)	F2
Kovacs	2014	Canada	Empagliflozin10mg:165 Empagliflozin25mg:168 Placebo:165	241/257	Empagliflozin10mg:54.7 Empagliflozin25mg:54.2 Placebo:54.6	Empagliflozin: 10/25mg once/daily	F2
Lavalle	2013	Mexico	Canagliflozin100mg:368 Canagliflozin300mg:367 Placebo:183	483/475	Canagliflozin100mg:55.5 Canagliflozin300mg:55.3 Placebo:55.3	Canagliflozin(100mg,300mg once/daily)	F1
Lee	2021	Korea	Dapagliflozin:41 Placebo:43	35/49	Dapagliflozin:59.7 Placebo:57.7	Dapagliflozin10mg once/daily	F1
List	2009	USA	Dapagliflozin2.5mg:59 Dapagliflozin5mg:58 Dapagliflozin10mg:47 Dapagliflozin20mg:59 Dapagliflozin50mg:56 Placebo:54	139/142	Dapagliflozin2.5mg:55 Dapagliflozin5mg:55 Dapagliflozin10mg:54 Dapagliflozin20mg:55 Dapagliflozin50mg:53 Placebo:53	Dapagliflozin (2.5mg, 1mg, 5mg,10mg, 20mg, 50mg once/daily)	F2
Stenlof	2013	USA	Canagliflozin100mg:195 Canagliflozin300mg:197 Placebo:192	258/326	Canagliflozin100mg:55.1 Canagliflozin300mg:55.3 Placebo:55.7	Canagliflozin(100mg,300mg once/daily)	F1
Strojek	2011	Poland	Dapagliflozin2.5mg:154 Dapagliflozin5mg:142 Dapagliflozin10mg:151 Placebo:145	285/307	Dapagliflozin2.5mg:59.9 Dapagliflozin5mg:60.2 Dapagliflozin10mg:58.9 Placebo:60.3	Dapagliflozin (2.5mg, 5mg,10mg, once/daily)	F2
Thiele	2021	Germany	Empagliflozin10mg:20 Placebo:22	18/34	Empagliflozin10mg:62 Placebo:62	Empagliflozin: 10mg once/daily	F1, F2
Wilding	2013	Netherlands	Dapagliflozin2.5mg:202 Dapagliflozin5mg:212 Dapagliflozin10mg:196 Placebo:197	382/228	Dapagliflozin2.5mg:59.8 Dapagliflozin5mg:59.3 Dapagliflozin10mg:59.3 Placebo:58.8	Dapagliflozin (2.5mg, 5mg,10mg, once/daily)	F2
Yale	2013		Canagliflozin100mg:90 Canagliflozin300mg:89 Placebo:90	163/106	Canagliflozin100mg:58 Canagliflozin300mg:58 Placebo:57	Canagliflozin(100mg,300mg once/daily)	F1; F2
Zinman	2015	USA	Empagliflozin10mg:2345 Empagliflozin25mg:2342 Placebo:2333	5016/2004	Empagliflozin10mg:63 Empagliflozin25mg:63.2 Placebo:63.2	Empagliflozin: 10/25mg once/daily	F1; F2

F1, Hemoglobin; F2, Hematocrit. NA: Not Applicable.

### 3.3 Results of NMA

#### 3.3.1 Hematocrit

**Figure 4:**
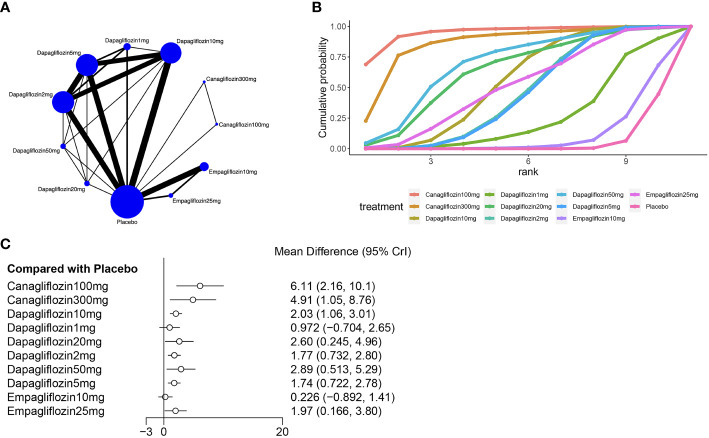
Meta-analysis of hematocrit **(A)**: Network diagram; Network graphs of all the drug agents included in the study. The width of the lines is proportional to the number of trials comparing each pair of treatments. The sizes of the circles are proportional to the number of trials using this intervention. **(B)**: surface under the cumulative ranking curve, **(C)**: Forest map of the effects drugs on the regulation of hematocrit).

Fifteen of the included (23, 25, 26, 28–31, 33, 34, 38, 45) studies reported hematocrit, as shown in **Figure 4**, and a closed loop was observed in the network diagram (**Figure 4A**). Therefore, the local inconsistency test was performed. The results suggested no difference in the direct, indirect and network comparisons between dapagliflozin 10 mg and dapagliflozin 1 mg, dapagliflozin 10mg and dapagliflozin 2mg, as well as between dapagliflozin 10mg and dapagliflozin 5mg, as shown in **Supplementary Figure 1**; **Supplementary Material 2**. Compared with placebo, canagliflozin 100mg [MD=6.11,95%CI(2.16, 10.1)], canagliflozin 300mg [MD=4.91,95%CI(1.05, 8.76)], dapagliflozin 10mg [MD=2.03,95%CI(1.06,3.01)], dapagliflozin 2mg [MD=1.77, 95%CI(0.732, 2.80)], dapagliflozin 50mg [MD=2.89, 95%CI(0.513, 5.29)], dapagliflozin 5mg [MD=1.74, 95%CI(0.772,2.78)], empagliflozin 25mg [MD=1.97,95%CI(0.166,3.80)], dapagliflozin 20 mg [MD = 2.6, 95% CI (0.245, 4.96)] increased hematocrit in patients with T2DM (**Figure 4C**), and the effect of canagliflozin 100 mg was better than that of dapagliflozin 10mg, dapagliflozin 1mg, dapagliflozin 2mg, dapagliflozin 5mg, and empagliflozin 10mg (**Supplementary Table 1** in **Supplementary Material 2**). The SUCRA of canagliflozin 100mg was the largest (91%), followed by canagliflozin 300mg (86%) and dapagliflozin 50mg (69%), and the SUCRA of placebo was the smallest (5%) (**Figure 4B**; **Table 3**).

**Table 3:** Overall probability ranking by SUCRA.

	Hemoglobin (%)	Hematocrit (%)
Canagliflozin100mg	87	95
Canagliflozin200mg	92	NA
Canagliflozin300mg	71	86
Dapagliflozin10mg	34	54
Empagliflozin10mg	27	11
Empagliflozin25mg	35	51
Placebo	4	5
Dapagliflozin1mg	NA	26
Dapagliflozin20mg	NA	64
Dapagliflozin2mg	NA	45
Dapagliflozin50mg	NA	69
Dapagliflozin5mg	NA	44

NA: Not Applicable.

#### 3.3.2 Hemoglobin

**Figure 5:**
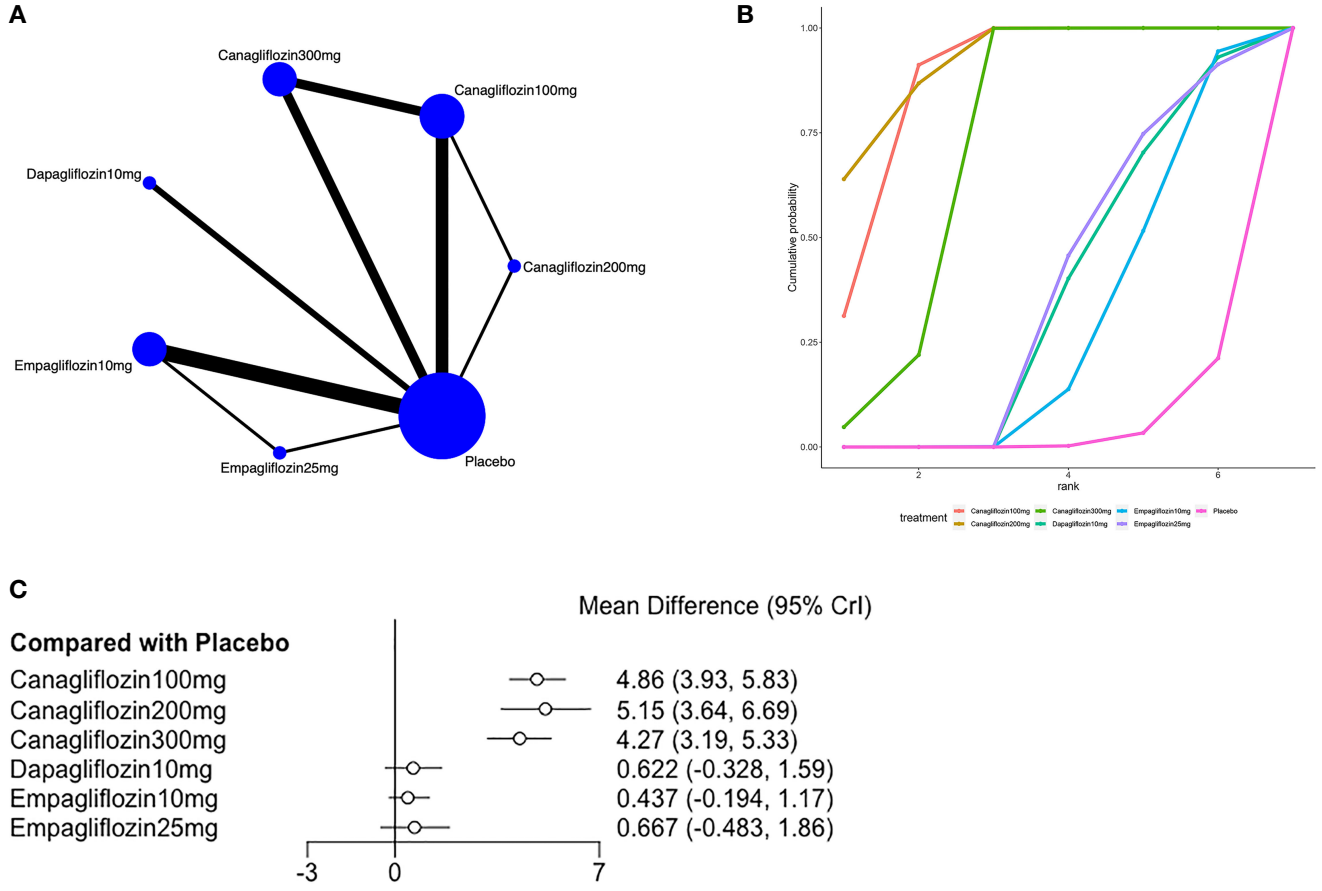
Meta-analysis of hemoglobin **(A)**: Network diagram; Network graphs of all the drug agents included in the study. The width of the lines is proportional to the number of trials comparing each pair of treatments. The sizes of the circles are proportional to the number of trials using this intervention. **(B)**: surface under the cumulative ranking curve, **(C)**: Forest map of the effects drugs on the regulation of hemoglobin).

Eleven of the included (23, 24, 27, 31, 33, 36, 37, 39, 41, 43, 44) articles reported hemoglobin, as shown in **Figure 5**. A closed loop was observed in the network diagram (**Figure 5A**), but the local inconsistency test was not performed due to the limited number of relevant studies. Compared with placebo, canagliflozin 100mg [MD=4.86,95%CI (3.93,5.83)], canagliflozin 200mg [MD=5.15,95%CI (3.64,6.69)], canagliflozin 300mg [MD = 4.27, 95% CI (3.19, 5.53)] increased hemoglobin in patients with T2DM (**Figure 5C**), but the effects of canagliflozin 100mg, canagliflozin 200mg, and canagliflozin 300mg showed no significant difference (**Supplementary Table 1** in **Supplementary Material 2**). The SUCRA of canagliflozin 200mg was the largest (92%), followed by canagliflozin 100mg (87%) and canagliflozin 300mg (71%), and the SUCRA of placebo was the smallest (4%) (**Figure 5B**; **Table 3**).

#### 3.3.3 Assessment of publication bias

We used funnel plots to evaluate the publication bias for hematocrit and hemoglobin, and the results suggested a high possibility of publication bias for hematocrit and hemoglobin (**Supplementary Figures 2, 3** in **Supplementary Material 2**).

#### 3.3.4 Sensitivity analysis

Sensitivity analysis was conducted on hemoglobin and hematocrit. The analysis results are shown in **Figures S4–6** and **Figures S7–14** in **Supplementary Material 2**, respectively.

## 4 Discussion

To our knowledge, this study is the first to evaluate the effects of different types and dosages of SGLT2i on the improvement of hematocrit and hemoglobin in patients with T2DM. A previous meta-analysis (15) indicated that SGLT2i increased hemoglobin and hematocrit levels compared with the control group, but it did not explore the dosage of SGLT2i in-depth and lacked a direct comparison between different interventions. We evaluated the effects of different dosages of SGLT2i, and this was an innovation of our research.

According to this study, canagliflozin 100mg [MD=6.11,95%CI (2.16, 10.1)] was most effective in increasing hematocrit, followed by canagliflozin 300mg [MD=4.91,95%CI (1.05, 8.76)] and dapagliflozin 50mg [MD=2.89, 95%CI (0.513, 5.29)], while canagliflozin 200mg [MD=5.15,95%CI (3.64,6.69)] was most effective in increasing hemoglobin, followed by canagliflozin 100mg [MD=4.86,95%CI (3.93,5.83)] and dapagliflozin 300mg [MD = 4.27, 95% CI (3.19, 5.53)]. The fact that SGLT2i increased hemoglobin and hematocrit could be partly explained by the unique role of SGLT2i in glucose excretion via the kidney, contributing to osmotic diuresis and leading to a significant increase in hemoglobin and hematocrit, which would help to lower blood pressure and relieve heart failure (46). Some studies have speculated that SGLT2i reduces ATP consumption through the Na+/K+ pump and alleviates the metabolic stress of renal proximal tubular epithelial cells (PTECs), thereby reducing hypoxia (47, 48) in the microenvironment around proximal renal tubules. This may allow myofibroblasts in the mesenchyme to reverse to some extent to erythropoietin-producing fibroblasts, enhancing the hematopoiesis and elevating hematocrit (49). Some other studies suggest that SGLT2i can prevent damage to renal mesenchymal cells by reducing the activity of the SGLT2 pathway on proximal tubular cells, and this will maintain an adequate erythropoietin level, and subsequently increase hemoglobin and hematocrit levels (50, 51). It has also been found in other studies that the increase of hematocrit may be closely related to the correction of sympathetic hyperfunction, leading to the reduction of cardiovascular mortality and the risk of hospitalization due to heart failure (52).

According to this study, canagliflozin showed the best effect, and this may be due to its dual inhibition of SGLT1 and SGLT2. Since SGLT1 is mainly expressed in the intestine, canagliflozin may change the intestinal environment or flora structure through the inhibition of intestinal SGLT1. Polidori et al. (53) found that canagliflozin decreased postprandial blood glucose and insulin by increasing urinary glucose excretion through the inhibition of renal SGLT2 and by delaying the use of oral glucose, and this might be related to the inhibition of intestinal SGLT1. Intestinal bacteria affect the iron absorption of the host, and iron intake will affect the virulence of bacteria. The host-intestinal bacteria interactions affect the systemic iron level and iron storage in different tissues and may therefore have an impact on hemoglobin or hematocrit (54). Although this study explored the differences between various doses, the top interventions showed no significant differences in effects as shown in the league table. This research provided an option for T2DM patients with anemia, but more research is needed to validate our findings on the choice of doses.

All the included studies clearly state the use of random method, and they are therefore of higher quality, indicating that our conclusions are reliable. In terms of hemoglobin, a low quality was rated, possibly due to the small number of included studies, and this may reduce the credibility of our conclusion. For hematocrit, a moderate quality was rated, and this may be caused by heterogeneity. The potential heterogeneity among studies may be due to the inconsistency of the study population or the inconsistency of the dose of the drugs used, but the potential heterogeneity did not affect our conclusions.

Although this study confirmed that SGLT2i could increase hematocrit and hemoglobin in patients with T2DM, the following limitations should be noted. First, hematocrit and hemoglobin were not analyzed as the primary outcomes in the included studies. Second, there was a greater heterogeneity among studies due to the different time for statistics of outcomes. Third, there was a greater heterogeneity among the treatment methods for the included patients.

## 5 Conclusion

Based on the existing studies, SGLT2i can improve hematocrit and hemoglobin in patients with T2DM, and canagliflozin 100mg is most effective in elevating hematocrit, while canagliflozin 200mg is most effective in elevating hemoglobin.

## Data availability statement

The original contributions presented in the study are included in the article/**Supplementary Material** Further inquiries can be directed to the corresponding author.

## Author contributions

YL: Data curation, Formal analysis, Investigation, Methodology, Software, Writing – original draft. RB: Data curation, Formal analysis, Investigation, Methodology, Software, Writing – original draft. WZ: Data curation, Formal analysis, Investigation, Software, Writing – original draft. GQ: Conceptualization, Funding acquisition, Resources, Supervision, Writing – review & editing.
