# Supplementary material for: Selective sodium-glucose cotransporter-2 inhibitors in the improvement of hemoglobin and hematocrit in patients with type 2 diabetes mellitus: a network meta-analysis
Source: Front Endocrinol (Lausanne). 2024 Feb 1;15:1333624. doi: 10.3389/fendo.2024.1333624 (PMC10867125; doi:10.3389/fendo.2024.1333624)
Supplement: Supplementary file 1 [file DataSheet_1.docx]

**Supplementary material 1: Search strategy**

Pubmed

((((("Sodium-Glucose Transporter 2 Inhibitors"[Mesh]) OR ((((((((((((((Sodium-Glucose Transporter 2 Inhibitors[Title/Abstract]) OR (Sodium Glucose Transporter 2 Inhibitors[Title/Abstract])) OR (SGLT-2 Inhibitors[Title/Abstract])) OR (SGLT 2 Inhibitors[Title/Abstract])) OR (SGLT2 Inhibitors[Title/Abstract])) OR (Sodium-Glucose Transporter 2 Inhibitor[Title/Abstract])) OR (Sodium Glucose Transporter 2 Inhibitor[Title/Abstract])) OR (SGLT2 Inhibitor[Title/Abstract])) OR (Inhibitor, SGLT2[Title/Abstract])) OR (Gliflozins[Title/Abstract])) OR (Gliflozin[Title/Abstract])) OR (SGLT-2 Inhibitor[Title/Abstract])) OR (Inhibitor, SGLT-2[Title/Abstract])) OR (SGLT 2 Inhibitor[Title/Abstract]))) OR ("Canagliflozin"[Mesh])) OR (((((((((((((((((((Canagliflozin[Title/Abstract]) OR (Invokana[Title/Abstract])) OR (Canagliflozin Hemihydrate[Title/Abstract])) OR (Canagliflozin, Anhydrous[Title/Abstract])) OR (Empagliflozin[Title/Abstract])) OR (BI 10773[Title/Abstract])) OR (BI10773[Title/Abstract])) OR (BI-10773[Title/Abstract])) OR (Jardiance[Title/Abstract])) OR (Dapagliflozin[Title/Abstract])) OR (Farxiga[Title/Abstract])) OR (Forxiga[Title/Abstract])) OR (BMS 512148[Title/Abstract])) OR (BMS512148[Title/Abstract])) OR (BMS-512148[Title/Abstract])) OR (sotagliflozin[Title/Abstract])) OR (LX4211[Title/Abstract])) OR (LX-4211[Title/Abstract])) OR (Epagliflozin[Title/Abstract]))) AND ((("Diabetes Mellitus, Type 2"[Mesh]) OR (((((((((((((((((((((((((((((Diabetes Mellitus, Type 2[Title/Abstract]) OR (Diabetes Mellitus, Noninsulin-Dependent[Title/Abstract])) OR (Diabetes Mellitus, Ketosis-Resistant[Title/Abstract])) OR (Diabetes Mellitus, Ketosis Resistant[Title/Abstract])) OR (Ketosis-Resistant Diabetes Mellitus[Title/Abstract])) OR (Diabetes Mellitus, Non Insulin Dependent[Title/Abstract])) OR (Diabetes Mellitus, Non-Insulin-Dependent[Title/Abstract])) OR (Non-Insulin-Dependent Diabetes Mellitus[Title/Abstract])) OR (Diabetes Mellitus, Stable[Title/Abstract])) OR (Stable Diabetes Mellitus[Title/Abstract])) OR (Diabetes Mellitus, Type II[Title/Abstract])) OR (NIDDM[Title/Abstract])) OR (Diabetes Mellitus, Noninsulin Dependent[Title/Abstract])) OR (Diabetes Mellitus, Maturity-Onset[Title/Abstract])) OR (Diabetes Mellitus, Maturity Onset[Title/Abstract])) OR (Maturity-Onset Diabetes Mellitus[Title/Abstract])) OR (Maturity Onset Diabetes Mellitus[Title/Abstract])) OR (MODY[Title/Abstract])) OR (Diabetes Mellitus, Slow-Onset[Title/Abstract])) OR (Diabetes Mellitus, Slow Onset[Title/Abstract])) OR (Slow-Onset Diabetes Mellitus[Title/Abstract])) OR (Type 2 Diabetes Mellitus[Title/Abstract])) OR (Noninsulin-Dependent Diabetes Mellitus[Title/Abstract])) OR (Noninsulin Dependent Diabetes Mellitus[Title/Abstract])) OR (Maturity-Onset Diabetes[Title/Abstract])) OR (Diabetes, Maturity-Onset[Title/Abstract])) OR (Maturity Onset Diabetes[Title/Abstract])) OR (Type 2 Diabetes[Title/Abstract])) OR (Diabetes, Type 2[Title/Abstract]))) OR (((Diabetes Mellitus, Adult-Onset[Title/Abstract]) OR (Adult-Onset Diabetes Mellitus[Title/Abstract])) OR (Diabetes Mellitus, Adult Onset[Title/Abstract])))) AND ((((("Hemoglobins"[Mesh]) OR (((((Hemoglobins[Title/Abstract]) OR (Hemoglobin[Title/Abstract])) OR (Eryhem[Title/Abstract])) OR (Ferrous Hemoglobin[Title/Abstract])) OR (Hemoglobin, Ferrous[Title/Abstract]))) OR ("Hematocrit"[Mesh])) OR (((((((((((((((Hematocrits[Title/Abstract]) OR (Hematocrit[Title/Abstract])) OR (Packed Red-Cell Volume[Title/Abstract])) OR (Packed Red Cell Volume[Title/Abstract])) OR (Packed Red-Cell Volumes[Title/Abstract])) OR (Red-Cell Volume, Packed[Title/Abstract])) OR (Red-Cell Volumes, Packed[Title/Abstract])) OR (Volume, Packed Red-Cell[Title/Abstract])) OR (Volumes, Packed Red-Cell[Title/Abstract])) OR (Erythrocyte Volume, Packed[Title/Abstract])) OR (Erythrocyte Volumes, Packed[Title/Abstract])) OR (Packed Erythrocyte Volume[Title/Abstract])) OR (Packed Erythrocyte Volumes[Title/Abstract])) OR (Volume, Packed Erythrocyte[Title/Abstract])) OR (Volumes, Packed Erythrocyte[Title/Abstract]))) OR ("Erythropoietin"[Mesh]))

Cochrane

ID Search Hits

#1 MeSH descriptor: [Sodium-Glucose Transporter 2 Inhibitors] explode all trees 735

#2 (Sodium Glucose Transporter 2 Inhibitors):ti,ab,kw OR (SGLT-2 Inhibitors):ti,ab,kw OR (SGLT 2 Inhibitors):ti,ab,kw OR (SGLT2 Inhibitors):ti,ab,kw OR (Sodium-Glucose Transporter 2 Inhibitor):ti,ab,kw 1947

#3 (Sodium Glucose Transporter 2 Inhibitor):ti,ab,kw OR (SGLT2 Inhibitor):ti,ab,kw OR (Inhibitor, SGLT2):ti,ab,kw OR (Gliflozins):ti,ab,kw OR (Gliflozin):ti,ab,kw 1535

#4 (SGLT-2 Inhibitor):ti,ab,kw OR (Inhibitor, SGLT-2):ti,ab,kw OR (SGLT 2 Inhibitor):ti,ab,kw 308

#5 MeSH descriptor: [Canagliflozin] explode all trees 317

#6 (Invokana):ti,ab,kw OR (Canagliflozin Hemihydrate):ti,ab,kw OR (Canagliflozin, Anhydrous):ti,ab,kw OR (Empagliflozin):ti,ab,kw OR (BI 10773):ti,ab,kw 1742

#7 (BI10773):ti,ab,kw OR (BI-10773):ti,ab,kw OR (Jardiance):ti,ab,kw OR (Dapagliflozin):ti,ab,kw OR (Farxiga):ti,ab,kw 2021

#8 (Forxiga):ti,ab,kw OR (BMS 512148):ti,ab,kw OR (BMS512148):ti,ab,kw OR (BMS-512148):ti,ab,kw OR (sotagliflozin):ti,ab,kw 281

#9 (LX4211):ti,ab,kw OR (LX-4211):ti,ab,kw OR (Epagliflozin):ti,ab,kw 45

#10 #1 OR #2 OR #3 OR #4 OR #5 OR #6 OR #7 OR #8 OR #9 4827

#11 MeSH descriptor: [Diabetes Mellitus, Type 2] explode all trees 23168

#12 (Diabetes Mellitus, Noninsulin-Dependent):ti,ab,kw OR (Diabetes Mellitus, Ketosis-Resistant):ti,ab,kw OR (Diabetes Mellitus, Ketosis Resistant):ti,ab,kw OR (Ketosis-Resistant Diabetes Mellitus):ti,ab,kw OR (Diabetes Mellitus, Non Insulin Dependent):ti,ab,kw 21901

#13 (Diabetes Mellitus, Non-Insulin-Dependent):ti,ab,kw OR (Non-Insulin-Dependent Diabetes Mellitus):ti,ab,kw OR (Diabetes Mellitus, Stable):ti,ab,kw OR (Stable Diabetes Mellitus):ti,ab,kw OR (Diabetes Mellitus, Type II):ti,ab,kw 27034

#14 (NIDDM):ti,ab,kw OR (Diabetes Mellitus, Noninsulin Dependent):ti,ab,kw OR (Diabetes Mellitus, Maturity-Onset):ti,ab,kw OR (Diabetes Mellitus, Maturity Onset):ti,ab,kw OR (Maturity-Onset Diabetes Mellitus):ti,ab,kw 1661

#15 (Maturity Onset Diabetes Mellitus):ti,ab,kw OR (MODY):ti,ab,kw OR (Diabetes Mellitus, Slow-Onset):ti,ab,kw OR (Diabetes Mellitus, Slow Onset):ti,ab,kw OR (Slow-Onset Diabetes Mellitus):ti,ab,kw 159

#16 (Type 2 Diabetes Mellitus):ti,ab,kw OR (Noninsulin-Dependent Diabetes Mellitus):ti,ab,kw OR (Noninsulin Dependent Diabetes Mellitus):ti,ab,kw OR (Maturity-Onset Diabetes):ti,ab,kw OR (Diabetes, Maturity-Onset):ti,ab,kw 50534

#17 (Maturity Onset Diabetes):ti,ab,kw OR (Type 2 Diabetes):ti,ab,kw OR (Diabetes, Type 2):ti,ab,kw OR (Diabetes Mellitus, Adult-Onset):ti,ab,kw OR (Adult-Onset Diabetes Mellitus):ti,ab,kw 57540

#18 (Diabetes Mellitus, Adult Onset):ti,ab,kw 1695

#19 #11 OR #12 OR #13 OR #14 OR #15 OR #16 OR #17 OR #18 61803

#20 MeSH descriptor: [Hemoglobins] explode all trees 11630

#21 (Hemoglobin):ti,ab,kw OR (Eryhem):ti,ab,kw OR (Ferrous Hemoglobin):ti,ab,kw OR (Hemoglobin, Ferrous):ti,ab,kw 44198

#22 MeSH descriptor: [Hematocrit] explode all trees 2082

#23 (Hematocrits):ti,ab,kw OR (Packed Red-Cell Volume):ti,ab,kw OR (Packed Red Cell Volume):ti,ab,kw OR (Packed Red-Cell Volumes):ti,ab,kw OR (Red-Cell Volume, Packed):ti,ab,kw 291

#24 (Red-Cell Volumes, Packed):ti,ab,kw OR (Volume, Packed Red-Cell):ti,ab,kw OR (Volumes, Packed Red-Cell):ti,ab,kw OR (Erythrocyte Volume, Packed):ti,ab,kw OR (Erythrocyte Volumes, Packed):ti,ab,kw 209

#25 (Packed Erythrocyte Volume):ti,ab,kw OR (Packed Erythrocyte Volumes):ti,ab,kw OR (Volume, Packed Erythrocyte):ti,ab,kw OR (Volumes, Packed Erythrocyte):ti,ab,kw 173

#26 MeSH descriptor: [Erythropoietin] explode all trees 2456

#27 #20 OR #21 OR #22 OR #23 OR #24 OR #25 OR #26 47715

#28 #10 AND #19 AND # 27 447

Web of science

| # |  | Result |
| --- | --- | --- |
| 1 | TS=(Sodium-Glucose Transporter 2 Inhibitors ) OR TS=(Sodium Glucose Transporter 2 Inhibitors) OR TS=(SGLT-2 Inhibitors) OR TS=(SGLT 2 Inhibitors) OR TS=(SGLT2 Inhibitors) OR TS=(Sodium-Glucose Transporter 2 Inhibitor) OR TS=(Sodium Glucose Transporter 2 Inhibitor) OR TS=(SGLT2 Inhibitor) OR TS=(Inhibitor, SGLT2) OR TS=(Gliflozins) OR TS=(Gliflozin) OR TS=(SGLT-2 Inhibitor) OR TS=(Inhibitor, SGLT-2) OR TS=(SGLT 2 Inhibitor) OR TS=(Canagliflozin) OR TS=(Invokana) OR TS=(Canagliflozin Hemihydrate) OR TS=(Canagliflozin, Anhydrous) OR TS=(Empagliflozin) OR TS=(BI 10773) OR TS=(BI10773) OR TS=(BI-10773) OR TS=(Jardiance) OR TS=(Dapagliflozin) OR TS=(Farxiga) OR TS=(Forxiga) OR TS=(BMS 512148) OR TS=(BMS512148) OR TS=(BMS-512148) OR TS=(sotagliflozin) OR TS=(LX4211) OR TS=(LX-4211) OR TS=(Epagliflozin) | 14448 |
| 2 | TS=(Diabetes Mellitus, Type 2 ) OR TS=(Diabetes Mellitus, Noninsulin-Dependent) OR TS=(Diabetes Mellitus, Ketosis-Resistant) OR TS=(Diabetes Mellitus, Ketosis Resistant) OR TS=(Ketosis-Resistant Diabetes Mellitus) OR TS=(Diabetes Mellitus, Non Insulin Dependent) OR TS=(Diabetes Mellitus, Non-Insulin-Dependent) OR TS=(Non-Insulin-Dependent Diabetes Mellitus) OR TS=(Diabetes Mellitus, Stable) OR TS=(Stable Diabetes Mellitus) OR TS=(Diabetes Mellitus, Type II) OR TS=(NIDDM) OR TS=(Diabetes Mellitus, Noninsulin Dependent) OR TS=(Diabetes Mellitus, Maturity-Onset) OR TS=(Diabetes Mellitus, Maturity Onset) OR TS=(Maturity-Onset Diabetes Mellitus) OR TS=(Maturity Onset Diabetes Mellitus) OR TS=(MODY) OR TS=(Diabetes Mellitus, Slow-Onset) OR TS=(Diabetes Mellitus, Slow Onset) OR TS=(Slow-Onset Diabetes Mellitus) OR TS=(Type 2 Diabetes Mellitus) OR TS=(Noninsulin-Dependent Diabetes Mellitus) OR TS=(Noninsulin Dependent Diabetes Mellitus) OR TS=(Maturity-Onset Diabetes) OR TS=(Diabetes, Maturity-Onset) OR TS=(Maturity Onset Diabetes) OR TS=(Type 2 Diabetes) OR TS=(Diabetes, Type 2) OR TS=(Diabetes Mellitus, Adult-Onset) OR TS=(Adult-Onset Diabetes Mellitus) OR TS=(Diabetes Mellitus, Adult Onset) | 291518 |
| 3 | TS=(Hemoglobins) OR TS=(Hemoglobin) OR TS=(Eryhem) OR TS=(Ferrous Hemoglobin) OR TS=(Hemoglobin, Ferrous) OR TS=(Hematocrit) OR TS=(Hematocrits) OR TS=(Packed Red-Cell Volume) OR TS=(Packed Red Cell Volume) OR TS=(Packed Red-Cell Volumes) OR TS=(Red-Cell Volume, Packed) OR TS=(Red-Cell Volumes, Packed) OR TS=(Volume, Packed Red-Cell) OR TS=(Volumes, Packed Red-Cell) OR TS=(Erythrocyte Volume, Packed) OR TS=(Erythrocyte Volumes, Packed) OR TS=(Packed Erythrocyte Volume) OR TS=(Packed Erythrocyte Volumes) OR TS=(Volume, Packed Erythrocyte) OR TS=(Volumes, Packed Erythrocyte) OR TS=(Erythropoietin) | 257010 |
| 4 | #1 AND #2 AND #3 | 1110 |

Embase

| No. | Query | Results |
| --- | --- | --- |
| #91 | #90 AND 'randomized controlled trial'/de | 1085 |
| #90 | #34 AND #67 AND #89 | 6323 |
| #89 | #68 OR #69 OR #70 OR #71 OR #72 OR #73 OR #74 OR #75 OR #76 OR #77 OR #78 OR #79 OR #80 OR #81 OR #82 OR #83 OR #84 OR #85 OR #86 OR #87 OR #88 | 595954 |
| #88 | 'erythropoietin'/exp | 40890 |
| #87 | 'volumes, packed erythrocyte':ab,ti | 0 |
| #86 | 'volume, packed erythrocyte':ab,ti | 0 |
| #85 | 'packed erythrocyte volumes':ab,ti | 2 |
| #84 | 'packed erythrocyte volume':ab,ti | 14 |
| #83 | 'erythrocyte volumes, packed':ab,ti | 0 |
| #82 | 'erythrocyte volume, packed':ab,ti | 0 |
| #81 | 'volumes, packed red-cell':ab,ti | 0 |
| #80 | 'volume, packed red-cell':ab,ti | 1 |
| #79 | 'red-cell volumes, packed':ab,ti | 0 |
| #78 | 'red-cell volume, packed':ab,ti | 0 |
| #77 | 'packed red-cell volumes':ab,ti | 18 |
| #76 | 'packed red cell volume':ab,ti | 116 |
| #75 | 'packed red-cell volume':ab,ti | 116 |
| #74 | 'hematocrits':ab,ti | 1929 |
| #73 | 'hematocrit'/exp | 83503 |
| #72 | 'hemoglobin, ferrous':ab,ti | 10 |
| #71 | 'ferrous hemoglobin':ab,ti | 54 |
| #70 | 'eryhem':ab,ti | 3 |
| #69 | 'hemoglobin':ab,ti | 218981 |
| #68 | 'hemoglobin'/exp | 466720 |
| #67 | #35 OR #36 OR #37 OR #38 OR #39 OR #40 OR #41 OR #42 OR #43 OR #44 OR #45 OR #46 OR #47 OR #48 OR #49 OR #50 OR #51 OR #52 OR #53 OR #54 OR #55 OR #56 OR #57 OR #58 OR #59 OR #60 OR #61 OR #62 OR #63 OR #64 OR #65 OR #66 | 380394 |
| #66 | 'diabetes mellitus, adult onset':ab,ti | 3 |
| #65 | 'adult-onset diabetes mellitus':ab,ti | 190 |
| #64 | 'diabetes mellitus, adult-onset':ab,ti | 3 |
| #63 | 'diabetes, type 2':ab,ti | 2840 |
| #62 | 'type 2 diabetes':ab,ti | 246822 |
| #61 | 'maturity onset diabetes':ab,ti | 3000 |
| #60 | 'diabetes, maturity-onset':ab,ti | 55 |
| #59 | 'maturity-onset diabetes':ab,ti | 3000 |
| #58 | 'noninsulin dependent diabetes mellitus':ab,ti | 1057 |
| #57 | 'noninsulin-dependent diabetes mellitus':ab,ti | 1057 |
| #56 | 'type 2 diabetes mellitus':ab,ti | 85887 |
| #55 | 'slow-onset diabetes mellitus':ab,ti | 0 |
| #54 | 'diabetes mellitus, slow onset':ab,ti | 1 |
| #53 | 'diabetes mellitus, slow-onset':ab,ti | 1 |
| #52 | 'mody':ab,ti | 2763 |
| #51 | 'maturity onset diabetes mellitus':ab,ti | 172 |
| #50 | 'maturity-onset diabetes mellitus':ab,ti | 172 |
| #49 | 'diabetes mellitus, maturity onset':ab,ti | 15 |
| #48 | 'diabetes mellitus, maturity-onset':ab,ti | 15 |
| #47 | 'diabetes mellitus, noninsulin dependent':ab,ti | 5 |
| #46 | 'niddm':ab,ti | 8102 |
| #45 | 'diabetes mellitus, type ii':ab,ti | 1381 |
| #44 | 'stable diabetes mellitus':ab,ti | 26 |
| #43 | 'diabetes mellitus, stable':ab,ti | 17 |
| #42 | 'non-insulin-dependent diabetes mellitus':ab,ti | 7862 |
| #41 | 'diabetes mellitus, non-insulin-dependent':ab,ti | 43 |
| #40 | 'diabetes mellitus, non insulin dependent':ab,ti | 43 |
| #39 | 'ketosis-resistant diabetes mellitus':ab,ti | 2 |
| #38 | 'diabetes mellitus, ketosis resistant':ab,ti | 0 |
| #37 | 'diabetes mellitus, ketosis-resistant':ab,ti | 0 |
| #36 | 'diabetes mellitus, noninsulin-dependent':ab,ti | 5 |
| #35 | 'non insulin dependent diabetes mellitus'/exp | 333847 |
| #34 | #1 OR #2 OR #3 OR #4 OR #5 OR #6 OR #7 OR #8 OR #9 OR #10 OR #11 OR #12 OR #13 OR #14 OR #15 OR #16 OR #17 OR #18 OR #19 OR #20 OR #21 OR #22 OR #23 OR #24 OR #25 OR #26 OR #27 OR #28 OR #29 OR #30 OR #31 OR #32 OR #33 | 25369 |
| #33 | 'epagliflozin':ab,ti | 1 |
| #32 | 'lx-4211':ab,ti | 8 |
| #31 | 'lx4211':ab,ti | 62 |
| #30 | 'sotagliflozin':ab,ti | 286 |
| #29 | 'bms-512148':ab,ti | 4 |
| #28 | 'bms512148':ab,ti | 6 |
| #27 | 'bms 512148':ab,ti | 4 |
| #26 | 'forxiga':ab,ti | 41 |
| #25 | 'farxiga':ab,ti | 34 |
| #24 | 'dapagliflozin':ab,ti | 4396 |
| #23 | 'jardiance':ab,ti | 65 |
| #22 | 'bi-10773':ab,ti | 27 |
| #21 | 'bi10773':ab,ti | 7 |
| #20 | 'bi 10773':ab,ti | 27 |
| #19 | 'empagliflozin':ab,ti | 4441 |
| #18 | 'canagliflozin, anhydrous':ab,ti | 0 |
| #17 | 'canagliflozin hemihydrate':ab,ti | 4 |
| #16 | 'invokana':ab,ti | 57 |
| #15 | 'canagliflozin'/exp | 5401 |
| #14 | 'sglt 2 inhibitor':ab,ti | 812 |
| #13 | 'inhibitor, sglt-2':ab,ti | 27 |
| #12 | 'sglt-2 inhibitor':ab,ti | 814 |
| #11 | 'gliflozin':ab,ti | 97 |
| #10 | 'gliflozins':ab,ti | 276 |
| #9 | 'inhibitor, sglt2':ab,ti | 102 |
| #8 | 'sglt2 inhibitor':ab,ti | 3483 |
| #7 | 'sodium glucose transporter 2 inhibitor':ab,ti | 64 |
| #6 | 'sodium-glucose transporter 2 inhibitor':ab,ti | 64 |
| #5 | 'sglt2 inhibitors':ab,ti | 5245 |
| #4 | 'sglt 2 inhibitors':ab,ti | 1673 |
| #3 | 'sglt-2 inhibitors':ab,ti | 1675 |
| #2 | 'sodium glucose transporter 2 inhibitors':ab,ti | 148 |
| #1 | 'sodium glucose cotransporter 2 inhibitor'/exp | 24497 |
