# Supplementary material for: Selective sodium-glucose cotransporter-2 inhibitors in the improvement of hemoglobin and hematocrit in patients with type 2 diabetes mellitus: a network meta-analysis
Source: Front Endocrinol (Lausanne). 2024 Feb 1;15:1333624. doi: 10.3389/fendo.2024.1333624 (PMC10867125; doi:10.3389/fendo.2024.1333624)
Supplement: Supplementary file 2 [file DataSheet_2.docx]

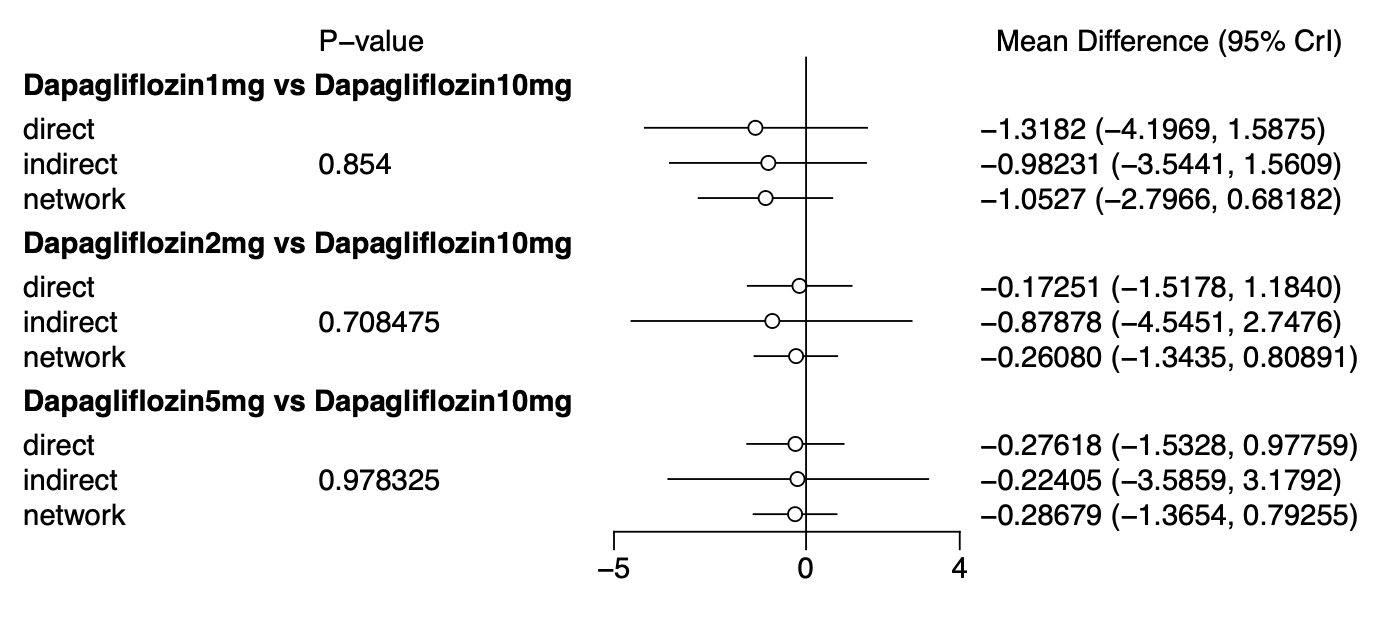


Figure S1: Local inconsistency test for Hematocrit


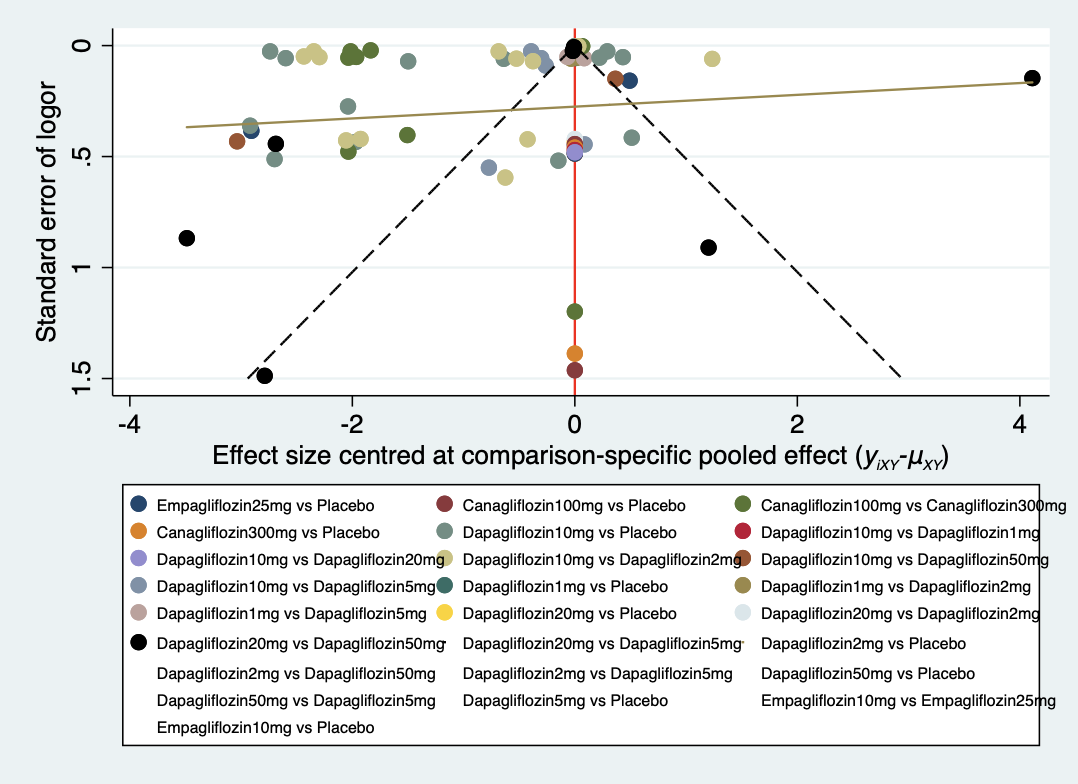


Figure S2 Funnel plot of Hematocrit


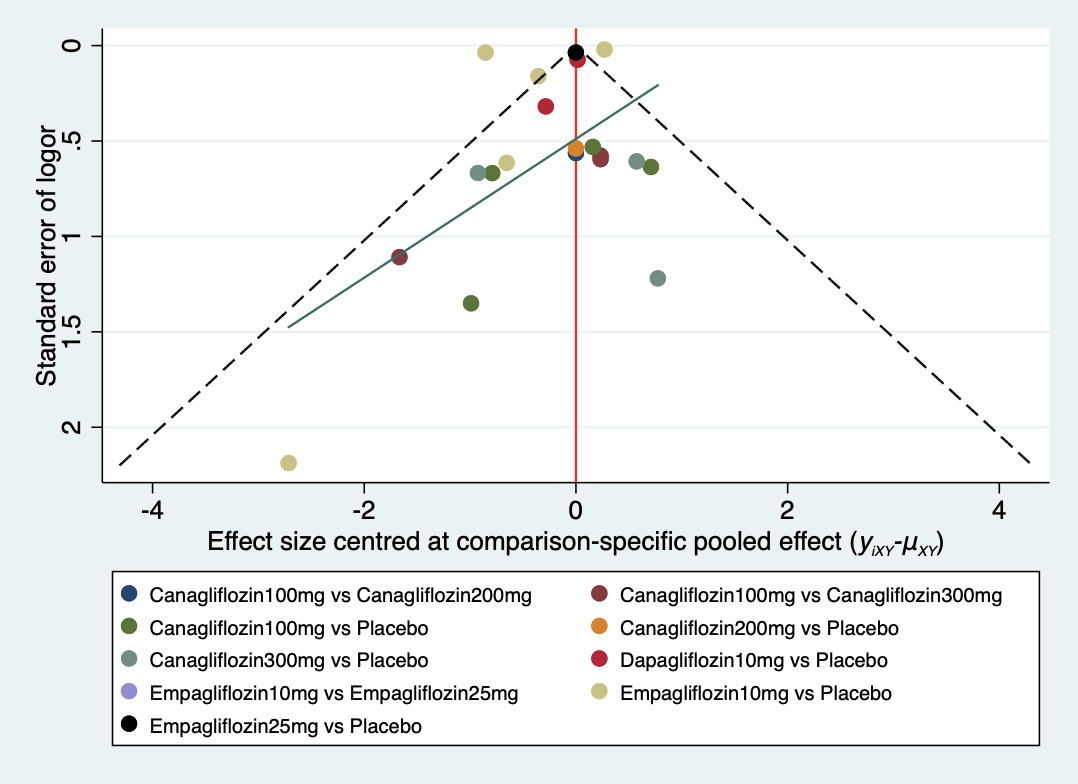


Figure S3 Funnel plot of Hemoglobin

Figure S4 Heterogeneity test for Hemoglobin


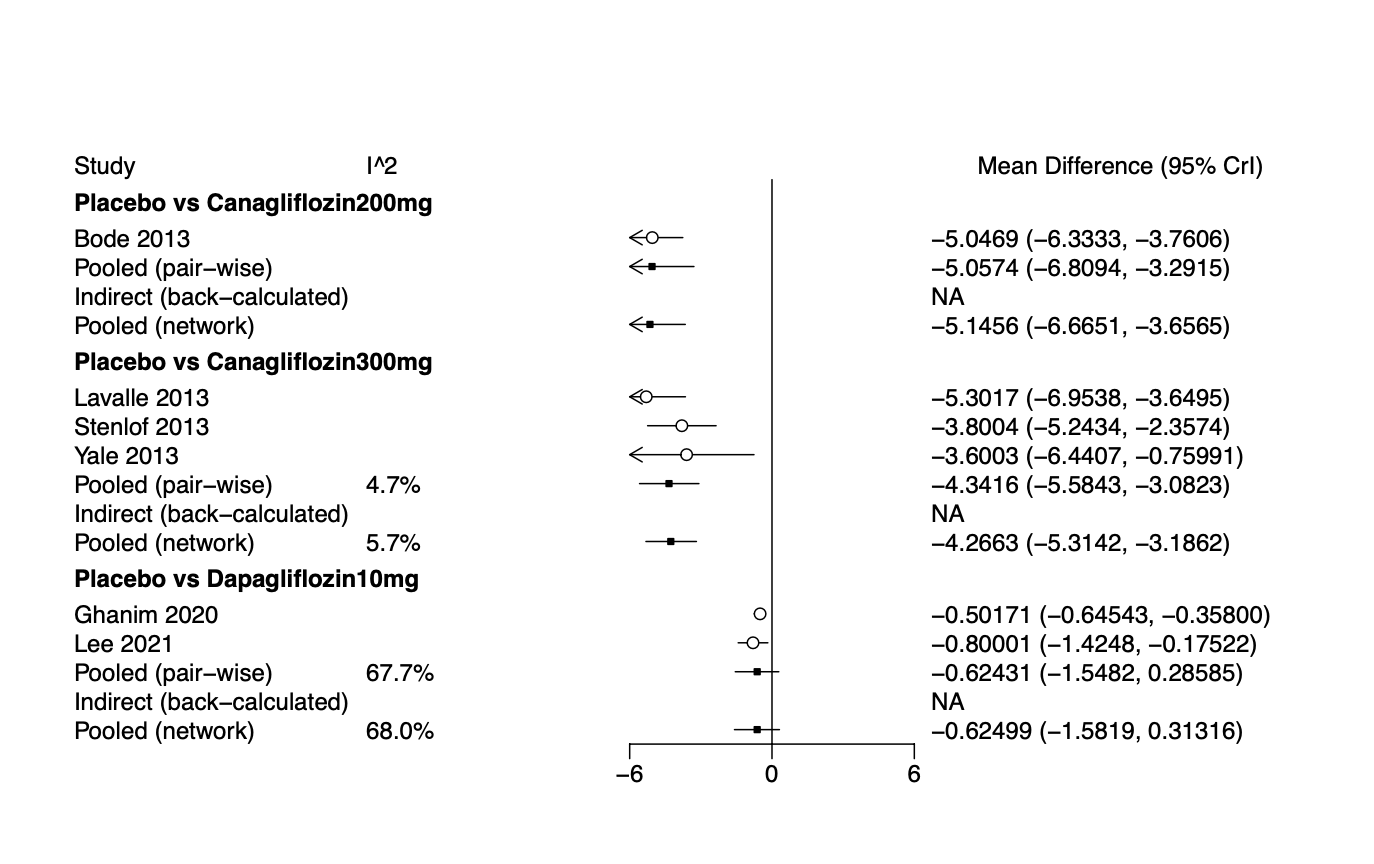


Figure S5 Heterogeneity test for Hemoglobin


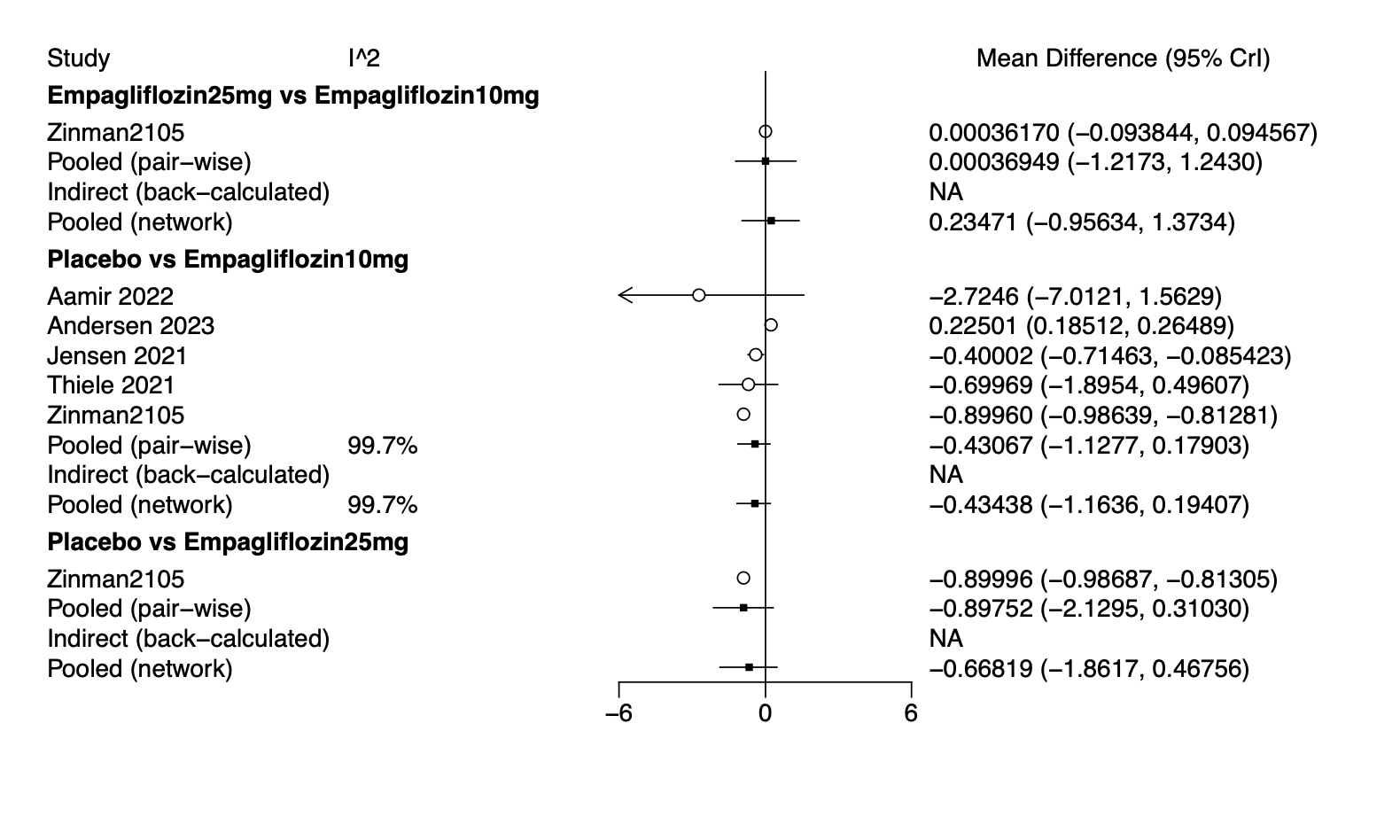


Figure S6 Heterogeneity test for Hemoglobin

Figure S7 Heterogeneity test for Hematocrit


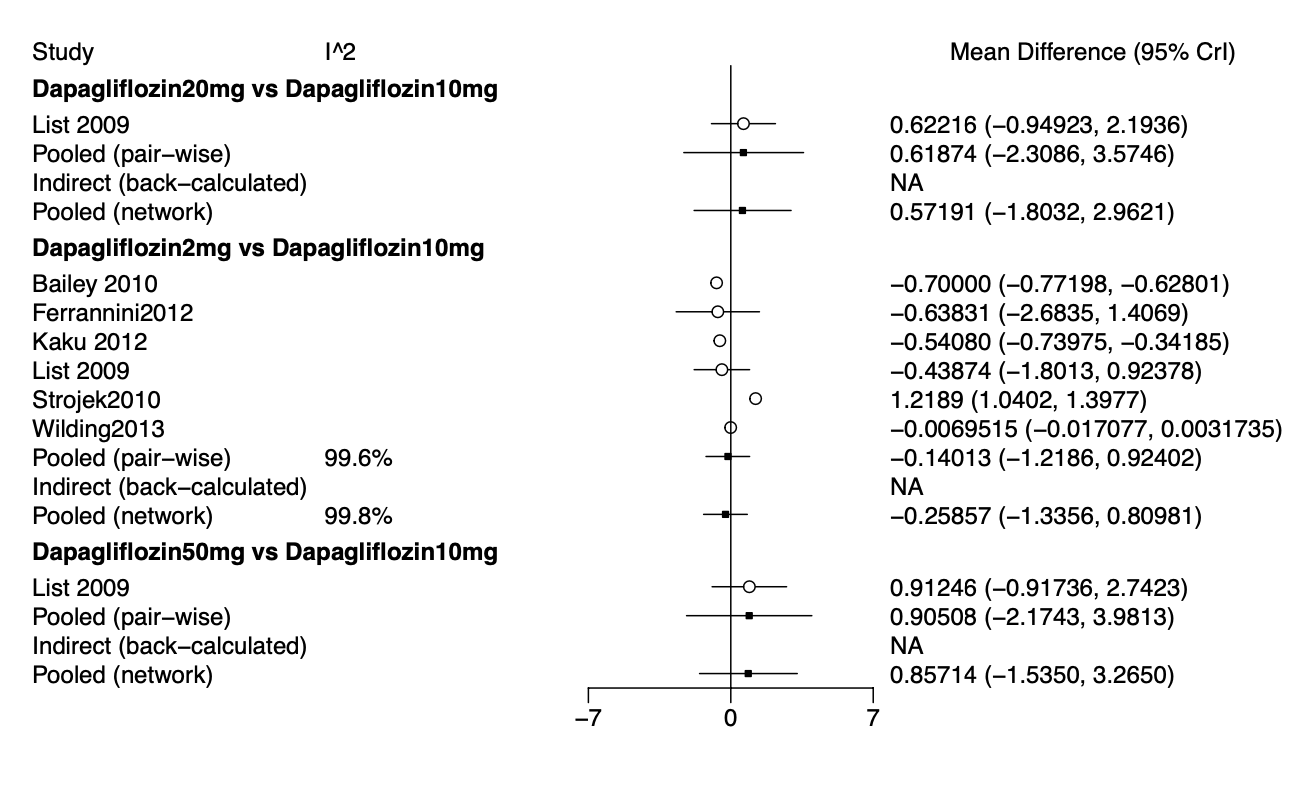


Figure S8 Heterogeneity test for Hematocrit


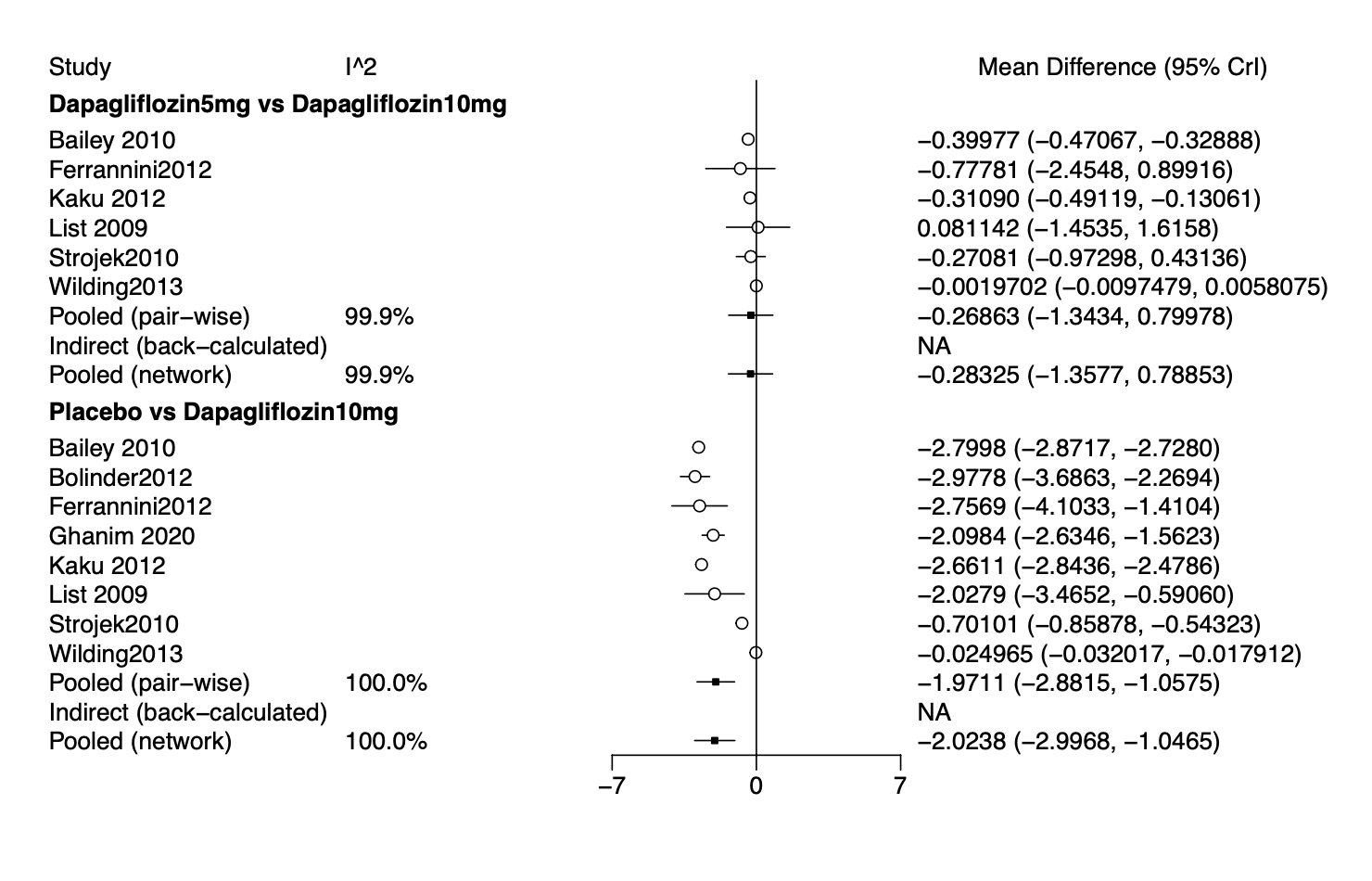


Figure S9 Heterogeneity test for Hematocrit


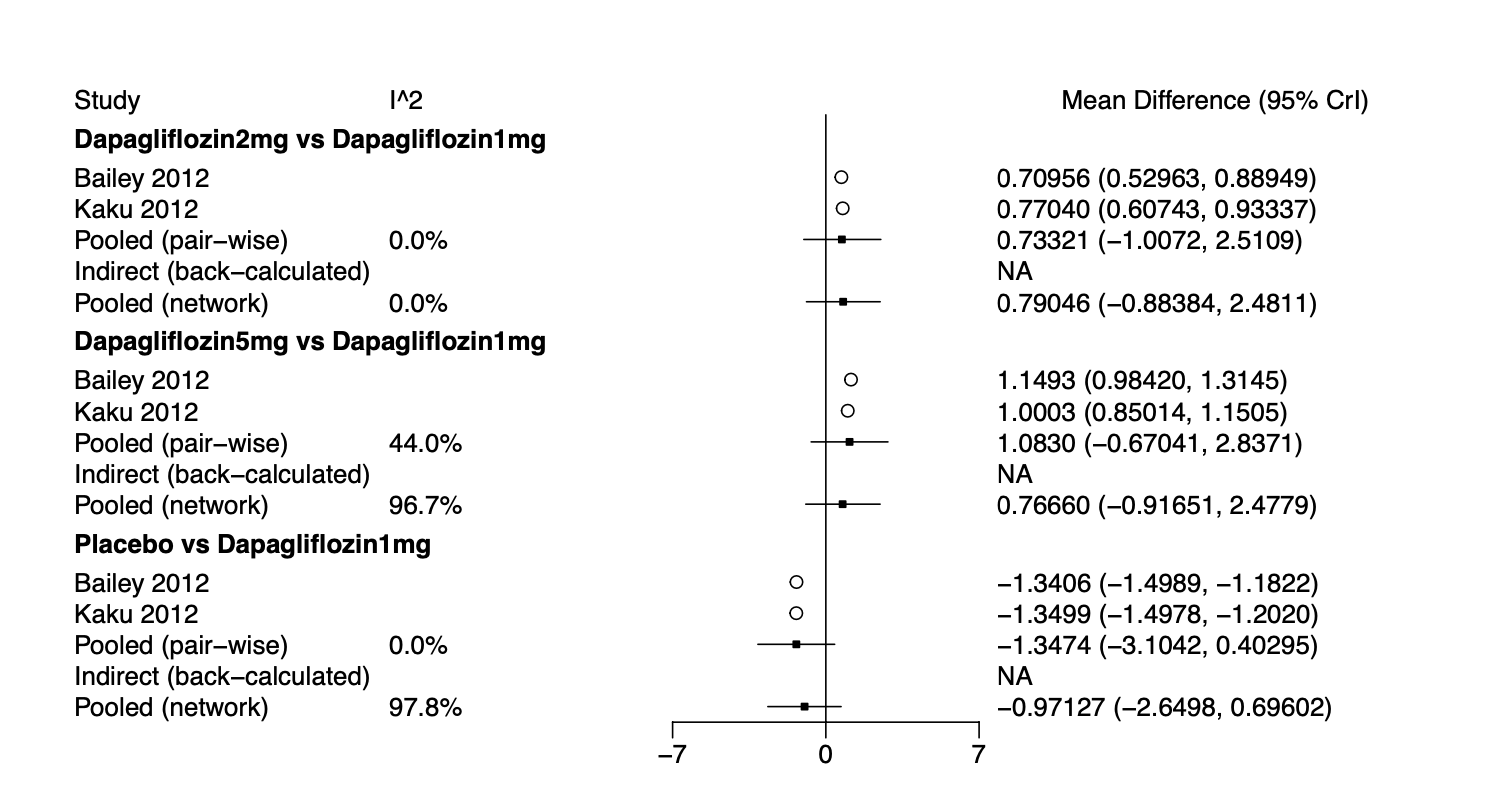


Figure S10 Heterogeneity test for Hematocrit


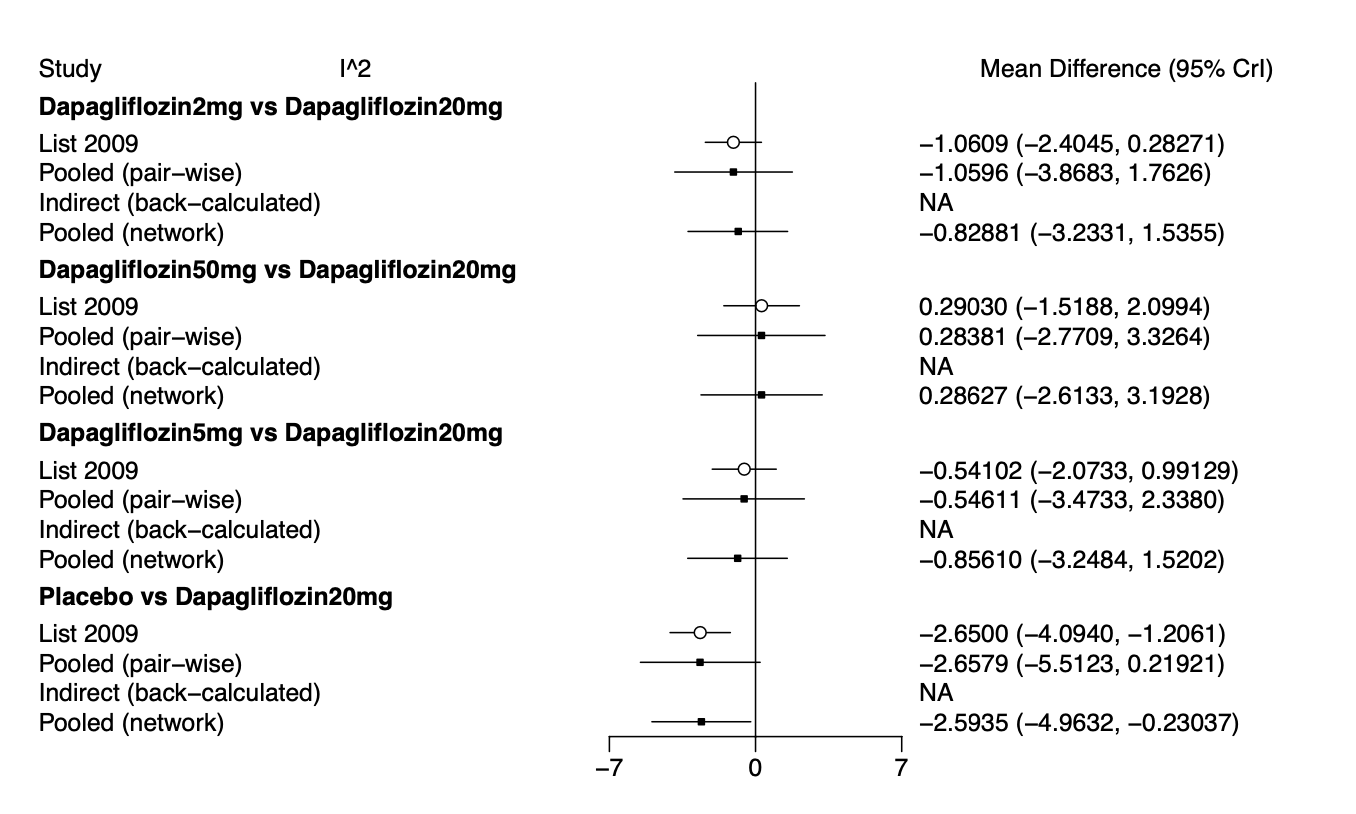


Figure S11 Heterogeneity test for Hematocrit


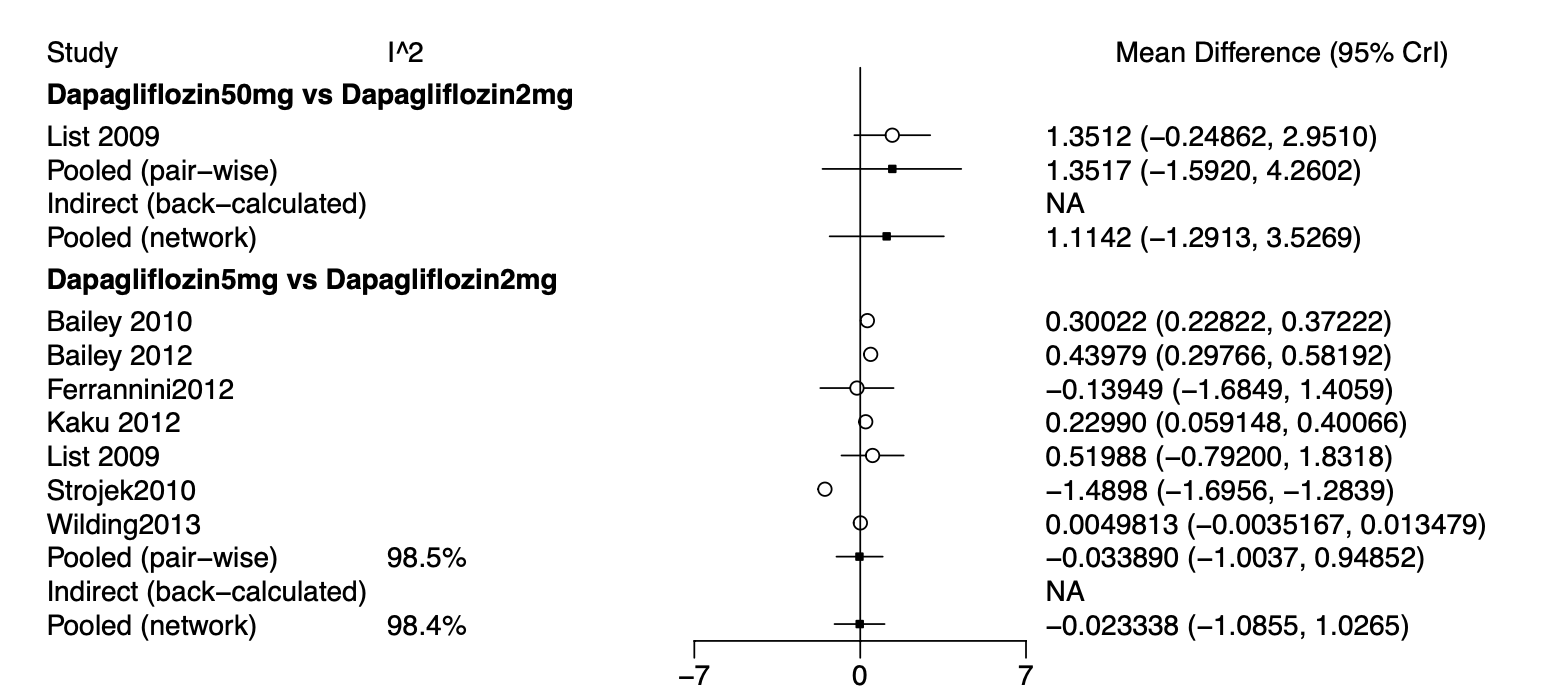


Figure S12 Heterogeneity test for Hematocrit


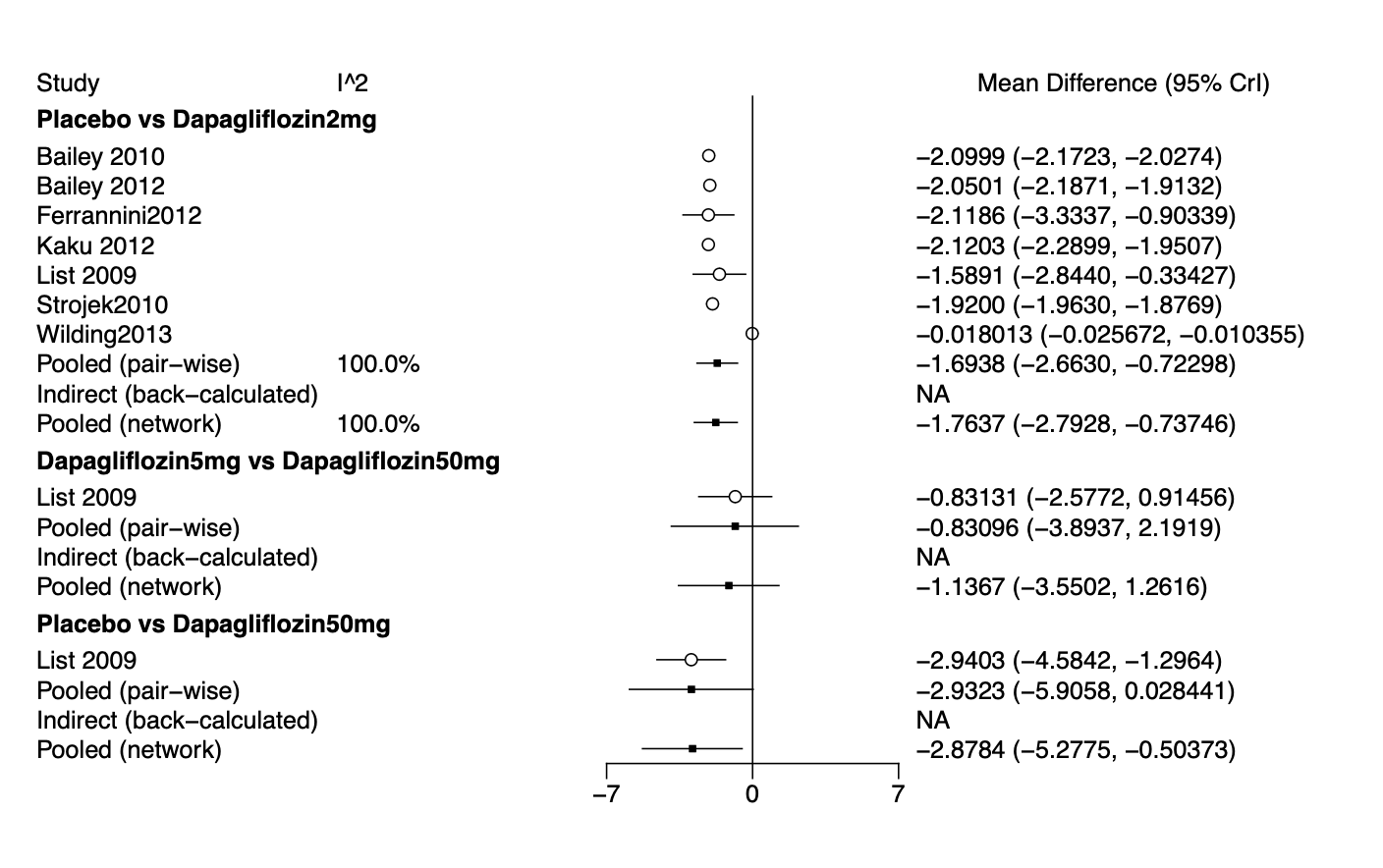


Figure S13 Heterogeneity test for Hematocrit


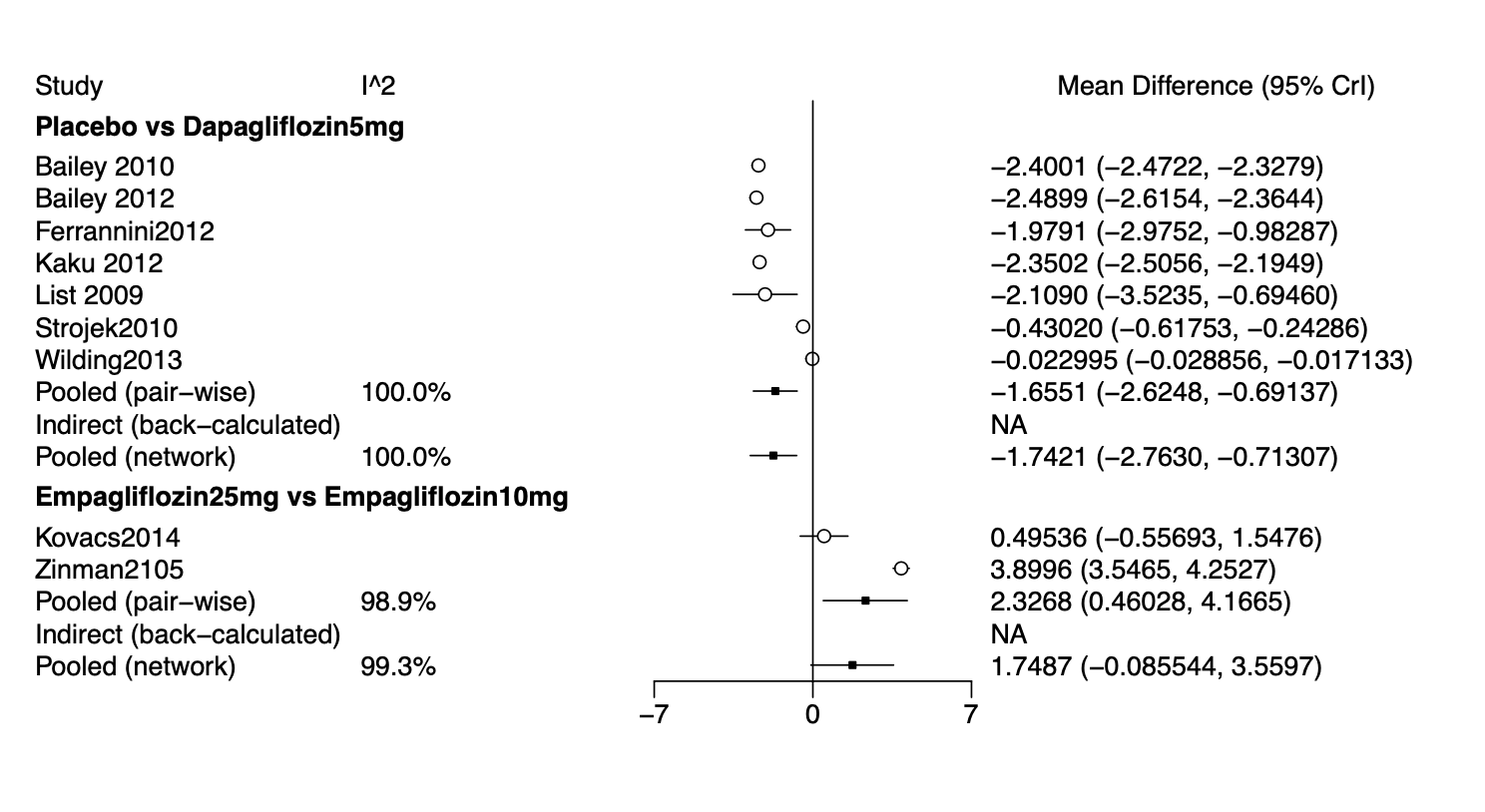


Figure S14 Heterogeneity test for Hematocrit

Table S1: League table

| **Hematocrit** | **MD 95%CI** | | | | | | | | | | | | **Hemoglobin** |
| --- | --- | --- | --- | --- | --- | --- | --- | --- | --- | --- | --- | --- | --- |
|  | Canagliflozin100mg | 0.29 (-1.26, 1.82) | -0.59 (-1.67, 0.42) | -4.24 (-5.6, -2.91) ^a^ | / | / | / | / | / | -4.42 (-5.57, -3.25)^a^ | -4.19 (-5.71, -2.71) ^a^ | -4.86 (-5.83, -3.93) ^a^ |  |
|  | / | Canagliflozin200mg | -0.88 (-2.65, 0.83) | -4.53 (-6.34, -2.73) ^a^ | / | / | / | / | / | -4.71 (-6.36, -3.03) ^a^ | -4.48 (-6.4, -2.56) ^a^ | -5.15 (-6.69, -3.64) |  |
|  | 1.2 (-2.43, 4.83) | / | Canagliflozin300mg | -3.65 (-5.06, -2.19) ^a^ | / | / | / | / | / | -3.83 (-5.05, -2.51) ^a^ | -3.6 (-5.15, -1.99) ^a^ | -4.27 (-5.33, -3.19) ^a^ |  |
|  | 4.08 (0, 8.15) ^a^ | / | 2.88 (-1.1, 6.84) | Dapagliflozin10mg | / | / | / | / | / | -0.18 (-1.32, 1.04) | 0.05 (-1.46, 1.58) | -0.62 (-1.59, 0.33) |  |
|  | 5.14 (0.83, 9.42) ^a^ | / | 3.94 (-0.26, 8.13) | 1.06 (-0.67, 2.8) | Dapagliflozin1mg | / | / | / | / | / | / | / |  |
|  | 3.5 (-1.1, 8.13) | / | 2.3 (-2.22, 6.84) | -0.57 (-2.93, 1.8) | -1.63 (-4.4, 1.15) | Dapagliflozin20mg | / | / | / | / | / | / |  |
|  | 4.34 (0.27, 8.42) ^a^ | / | 3.14 (-0.85, 7.14) | 0.27 (-0.81, 1.34) | -0.79 (-2.47, 0.89) | 0.84 (-1.54, 3.19) | Dapagliflozin2mg | / | / | / | / | / |  |
|  | 3.21 (-1.41, 7.84) | / | 2.02 (-2.52, 6.56) | -0.86 (-3.26, 1.54) | -1.93 (-4.71, 0.87) | -0.29 (-3.21, 2.61) | -1.13 (-3.53, 1.27) | Dapagliflozin50mg | / | / | / | / |  |
|  | 4.36 (0.29, 8.45) ^a^ | / | 3.17 (-0.82, 7.15) | 0.29 (-0.79, 1.36) | -0.77 (-2.46, 0.91) | 0.86 (-1.51, 3.23) | 0.02 (-1.03, 1.08) | 1.15 (-1.25, 3.55) | Dapagliflozin5mg | / | / | / |  |
|  | 5.87 (1.75, 9.98) ^a^ | / | 4.68 (0.63, 8.7) ^a^ | 1.8 (0.27, 3.28) ^a^ | 0.75 (-1.32, 2.75) | 2.38 (-0.27, 4.97) | 1.54 (-0.03, 3.06) | 2.66 (-0.01, 5.29) | 1.52 (-0.06, 3.04) | Empagliflozin10mg | 0.23 (-0.97, 1.38) | -0.44 (-1.17, 0.19) |  |
|  | 4.13 (-0.24, 8.48) | / | 2.93 (-1.35, 7.17) | 0.06 (-2, 2.11) | -1 (-3.48, 1.46) | 0.63 (-2.35, 3.6) | -0.2 (-2.3, 1.87) | 0.93 (-2.08, 3.91) | -0.23 (-2.32, 1.85) | -1.75 (-3.55, 0.09) | Empagliflozin25mg | -0.67 (-1.86, 0.48) |  |
|  | 6.11 (2.16, 10.07) ^a^ | / | 4.91 (1.05, 8.76) ^a^ | 2.03 (1.06, 3.01) ^a^ | 0.97 (-0.7, 2.65) | 2.6 (0.24, 4.96) ^a^ | 1.77 (0.73, 2.8) ^a^ | 2.89 (0.51, 5.29) ^a^ | 1.74 (0.72, 2.78) ^a^ | 0.23 (-0.89, 1.41) | 1.97 (0.17, 3.8) ^a^ | Placebo |  |

a means p<0.05
